# Double Ionization of Polycyclic Aromatic Nitrogen Heterocycles: Simulations on Phenanthridine

**DOI:** 10.1021/acsearthspacechem.5c00240

**Published:** 2026-01-30

**Authors:** Jorge H. C. Basilio, Germán Molpeceres, Wania Wolff, Miguel Pereira-Santaella, Ricardo R. Oliveira

**Affiliations:** † Physics Institute, 133645 Federal University of Rio de Janeiro, Rio de Janeiro, Rio de Janeiro 21941-909, Brazil; ‡ Departamento de Astrofísica Molecular, 16379 Instituto de Física Fundamental (IFF-CSIC), C/Serrano 121, Madrid 28006, Spain; § Chemistry Institute, 28125 Federal University of Rio de Janeiro, Rio de Janeiro 21941-909, Brazil

## Abstract

A relevant class of molecules is polycyclic aromatic nitrogen heterocycles (PANHs), which are important for the astrochemistry and biochemistry fields. Recently, three new nitrogen-bearing polycyclic aromatic molecules, cyanopyrene, cyanoacenaphthylene, and cyanocoronene, were detected in Taurus Molecular Cloud 1 (TMC-1) by radio astronomy, reinforcing the current relevance of this type of system. It is well-known that different charge states affect the structure and properties of these systems. Thus, we performed an automated search for structures and relative stabilities of the phenanthridine dication, a PANH molecular prototype. Also, computations were performed for phenanthrene dication for comparison and benchmarking reasons. Infrared spectra simulations were performed for both systems, and the main changes in spectral profile were discussed. We found that a huge isomerization reaction should occur after the double ionization process. The lowest-energy isomers of phenanthridine dication are protonated nitriles, i.e., the CNH group is formed bonded to an acenaphthene-like ring. Remarkably, this structure is very different when compared to PAH dications. The main bands in the IR spectra are located between 2000 cm^–1^ and 2500 cm^–1^ due to C–N stretching mode and around 3500 cm^–1^ due to N–H stretching mode. Finally, simulated dipole moments of low-energy isomers are very high, from 3.80 to 7.81 D, encouraging the detection of these species by radio astronomy.

## Graphical abstract

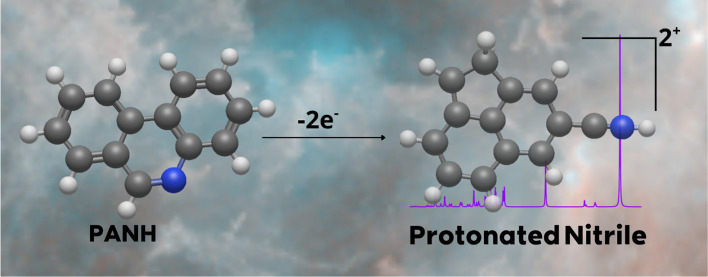

## 1 Introduction

It is well-known that polycyclic aromatic hydrocarbons (PAHs) are very abundant in interstellar (ISM) and circumstellar (CSM) media.^1^ This class of molecules is considered as aromatic infrared band (AIB) carriers. These bands are spanning in the 3–20 μm spectral range.^1–3^ The recent detection of pure aromatic and cyclic hydrocarbons in the Taurus Molecular Cloud 1 (TMC-1)^4–9^ reinforced the interest in the structural and spectroscopic properties of interstellar aromatic molecules. Particular research efforts were done on the study of infrared (IR) spectra of PAHs in order to correlate spectral signatures, AIBs, and molecular geometry variations or defect formation.^10–17^ Also, it is well-known that the charge state of PAHs has a huge influence on the structure and IR spectrum features.^10,17–22^

The ionization of PAHs can induce several isomerization reactions. A remarkable case is the benzene dication, whose global minimum (GM), i.e., the lowest energy structural arrangement of the molecules, isomer has an unusual pentagonal-pyramidal geometry, indicating an intense structural rearrangement after the second ionization step.^23–26^ For toluene (c-C_6_H_5_CH_3_), the situation is completely different. After the second ionization, only a hydrogen atom migration occurs from the CH_3_ to the six-membered ring at the meta position.^27^ Although simple aromatic molecules and small PAHs have been studied by several groups, the prediction of ionized low-energy isomer structures is not a trivial task. For instance, the cation and dication of naphthalene have a similar structure to the neutral species, but the trication and tetracation are completely different, with terminal C_3_H_2_ rings bonded to an open-chain C_4_H_4_ group.^18^ In the case of biphenyl cation and dication, the GM is composed of acenaphthene-like structures, where a C_2_ bridges a naphthalene-like backbone.^19^ For the phenanthrene dication, the GM features a three-fused ring (phenalene core) bonded to a methylene group, indicating again a huge isomerization after the second ionization process.^28^ Furthermore, the experimental IR spectrum is in good agreement with the GM-simulated one. It is worth noticing that dication with an anthracene-like structure is a low-lying energy isomer, differently from the phenanthrene-like one.^28^

In addition to PAHs, two important classes of molecules are polycyclic aromatic nitrogen heterocycles (PANHs)^29–31^ and polycyclic aromatic phosphorus heterocycles (PAPHs).^32,33^ Nitrogenated molecules are very relevant for molecular astrophysics due to the great number of recent nitrile detections (cyanopyrene—C_16_H_9_CN, cyanoacenaphthylene—C_12_H_7_CN, and cyanocoronene—C_24_H_11_CN),^8,34–42^ contributing to the increased interest in the formation and spectroscopic properties of nitrogen-bearing molecules.^29–31,43–55^ Another important feature of PANHs is the IR spectra intensity profiles. Some authors argue that PANHs and related cations can contribute to the interstellar emission band at 6.2 μm wavelength, thus reinforcing the importance of this class of molecules.^30,44,49,56^

The recent detection of PAH cyano derivatives was a cornerstone for the study of large nitrogen-bearing aromatic molecules.^8,40,42^ One great challenge is the strong dependence of properties and IR spectra with respect to molecular structure and charge states. Studies on prototype molecules assist in the understanding of structure-property correlations. For example, low-energy structures of aniline dication have a pyridine and azepine type of structure, different from the case of neutral and cation charge states.^57^ Other prototypes were investigated, such as pyridine,^31,54,56^ C_4_H_4_N,^50,58^ pyrrole,^58^ and benzonitrile.^43,56,59–61^ Although several efforts were made in order to understand the formation and spectroscopic properties of nitrogen-bearing molecules and related cations, there is no systematic study on the structure and IR spectra simulations of PANH ions at the dication state. At this charge state, the prediction of the geometry of low-energy isomers of aromatic molecules and, consequently, the spectroscopic profile in the IR region, is very challenging.^18,23–25,28,33^ Although no PANHs have been detected, these molecules can also be considered as markers for PAHs, in the same sense as the recently detected nitriles. The difficulty in detecting PANHs may be related to the fact that their dipole moment is smaller than that of cyanoaromatic compounds. In the case of PANHs, the nitrogen atom is part of the ring structure and does not form the CN group, decreasing the dipole moment compared to the detected aromatic molecules.

The aim of this work is the study of low-energy isomers and IR spectra simulations of a PANH prototype at the dication state, the phenanthridine molecule, whose neutral structure can be seen in Figure 1 (right). The phenanthrene dication (the neutral structure can be seen in Figure 1, left) was also investigated for benchmark purposes in order to validate our protocol. Beyond their astrochemical interest, both molecules in their neutral forms are well-known and studied on Earth. For instance, as early as 1950, Theobald and Schofield published a comprehensive review on the chemistry of phenanthridine and its derivatives.^62^ A direct comparison with phenanthrene results^28^ was done, highlighting the differences between PAHs and PANHs’ dication structure and IR features. To this end, an automated search for low-energy isomers of phenanthridine dication was performed, and IR spectra simulations at the Density Functional Theory (DFT) level were done. We note that our results regarding phenanthrene complement the work of Pereverzev and coworkers. We discuss the IR spectra in detail and also highlight the high dipole moment of the dications.^28^

**Figure 1:**
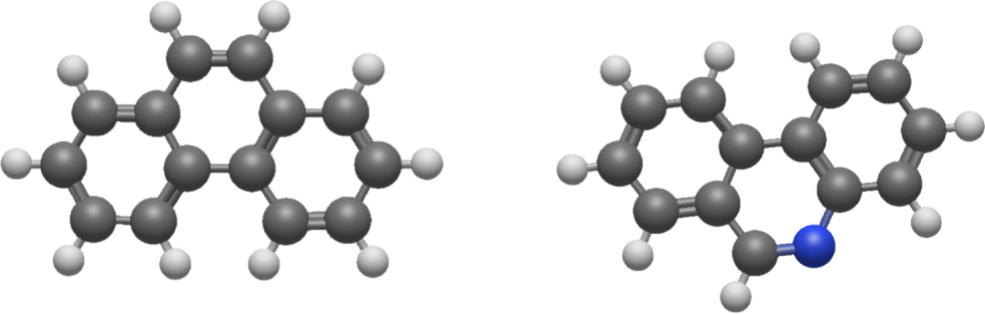
Neutral structures for phenanthrene (C_14_H_10_, left) and phenanthridine (C_13_H_9_N, right). Carbon atoms are gray, hydrogen atoms are white, and nitrogen atom is blue.

## 2 Computational Methods

Due to the elevated number of possible isomers of phenanthrene and phenanthridine molecules, with molecular formulas C_14_H_10_^2+^ and C_13_H_9_N^2+^, respectively, a human-guided search of low-lying isomers is completely unfeasible. In this work, we use an automated protocol to explore the potential energy surface of dicationic systems. In particular, we use the artificial force-induced reaction (AFIR) method implemented in the GRRM program.^63–66^

The AFIR method and, more specifically, the SC-AFIR variant, where SC means “single component” where the minimum search is started from an initial preoptimized structure, is an automatic reaction discovery method where the potential energy surface of the trial molecule is explored through successive force minimization upon application of forces between the fragments constituting the molecule, building a biased PES where kinetic barriers are submerged. As the name suggests, the methodology is suited to the identification of reaction paths between isomers, although in this work we only retrieved the discovered minima, as path and transition state optimizations significantly increase the number of energy and force evaluations. A detailed explanation of how the minimized AFIR function is constructed, applied between fragments, and the sampling algorithm is propagated is available in the review on the method by Maeda and Harabuchi.^67^

A single-component AFIR using two force simultaneous functions (SC-AFIR2) is used throughout, in both the singlet and triplet spin channels, although triplet structures were consistently found to be higher in energy and are not further discussed in the following. As an initial guess for a structure to start investigating the isomerizations, we used the structure of neutral phenanthrene and phenanthridine. The so-called collision energy in the AFIR method, γ, was set to 1000.0 kJ mol^–1^ for all calculations, ensuring the possibility of exploring high-energy regions in our exploration. Due to the number of energy evaluations required in our study (several tens of millions of single points per structure and spin channel), we did not look for transition states connecting the structures found in our search. To further reduce the computational cost, we set a stopping criterion in our search: stop the search if the last 350 runs did not yield any minima in the list of the lowest 100 already obtained in the simulation. Even with these considerations, our search yielded around 6000 minima in the case of phenanthrene and 5000 in the case of phenanthridine. The reason for studying phenanthrene in spite of only studying phenatridine was because it served as a benchmark system with known low-lying energy isomers.^28^ In this first step, the extended tight-binding (xtb) Hamiltonian, through GFN2-xTB parametrization^68^ implemented in the xTB software^69^ and interfaced with ORCA 5.0.3,^70^ was used for all calculations. It is important to note that the AFIR search performed in this work was designed to operate as a global minimum search. However, the same approach can be extended to characterize the full isomerization landscape of all structures identified here, effectively functioning as a reaction network generator. Such an undertaking, nevertheless, is extremely demanding as it would involve the identification of thousands of additional stationary points. Moreover, the associated kinetics to fully describe the ionization and posterior isomerization remain largely unknown, for instance, regarding energy redistribution, radiative emission, or the degree of ergodicity of the possible reactions, making this a formidable challenge.

A second step of geometry optimization was performed for the first 250 minimum structures obtained previously at the GFN2-xTB level of each molecule-multiplicity pair. In this case, the r^2^SCAN-3c (“Swiss army knife”)^71^ composite electronic-structure method was applied. Due to the mismatch between r^2^SCAN-3c and GFN2-xTB, not all structures converged. On average, 14% of the lowest 250 structures did not converge. Once the initial geometry optimization at the DFT level was performed, a third and last geometry optimization step was performed for the first 25 minimum structures at the Density Functional Theory level combined with the ωB97M-D4 functional (a modified version of ωB97M-V^72^ with DFT-D4 correction^73^) and the def2-TZVP basis set.^74^ Also, IR spectra simulations and zero-point energy (ZPE) calculations were performed at this level of theory. All frequencies were analyzed to confirm that all geometries represent a minimum on the PES. The IR spectra is obtained by broadening the normal modes obtained in the calculation of the analytic Hessian by Lorentzian fit with a FWHM (Full Width at Half Maximum) of 5.0 cm^–1^.

In order to correct the discrepancies arising from the harmonic approximation, we derived a set of scaling factors for the ωB97M-D4 functional. For this, we considered two experimental works where the normal vibrational modes of naphthalene^75^ and phenanthrene were measured and characterized.^76^ These systems were chosen as they are the closest structurally related molecules available in the literature to the systems studied in this work. To derive the scaling factors, we obtained the frequencies with the ωB97M-D4 functional for neutral naphthalene and phenanthrene and compared them to their respective experimentally active modes. The theoretical modes with zero or near-zero intensity were excluded from the comparison set. By applying the least-squares equation $\left(\lambda =\frac{\sum \left(w_{\mathrm{calc}}\cdot w_{\exp}\right)}{\sum {\left(w_{\mathrm{calc}}\right)}^{2}}\right)$ , we derived a dual-scaling factor set for two regions: (I) above 2000 cm^–1^ and (II) below 2000 cm^–1^. The values were λ_I_ = 0.956 and λ_II_ = 0.978. A similar approach was applied in a previous work.^18^ Moreover, anharmonic corrections and simulations were obtained through the VPT2 method.^77^

It is important to note that the present models for infrared spectra are specifically designed to reproduce the absorption features. Consequently, observational features seen in emission would require an appropriate conversion factor to ensure a proper comparison. Finally, in order to obtain more accurate electronic energies, single-point calculations were performed at the coupled cluster level using the DLPNO–CCSD­(T) approximation^78^ within the cc-pVTZ basis set.^79^ The final reported energies are the sum of ZPE from DFT (ωB97M-D4/def2-TZVP) with electronic energy from the coupled cluster method.

## 3 Results and Discussion

### 3.1 Low Energy Phenanthrene Dication Isomers

Following the identification of the low-energy isomers of phenanthrene (Figure 2), we will now discuss the formation of these structures for phenanthrene^2+^. The energies were organized such that the lowest-energy (GM candidate) is represented as 0.0 kcal mol^–1^, with the remaining candidates displaying their Δ*E* values in kcal mol^–1^ (relative energies).

**Figure 2:**
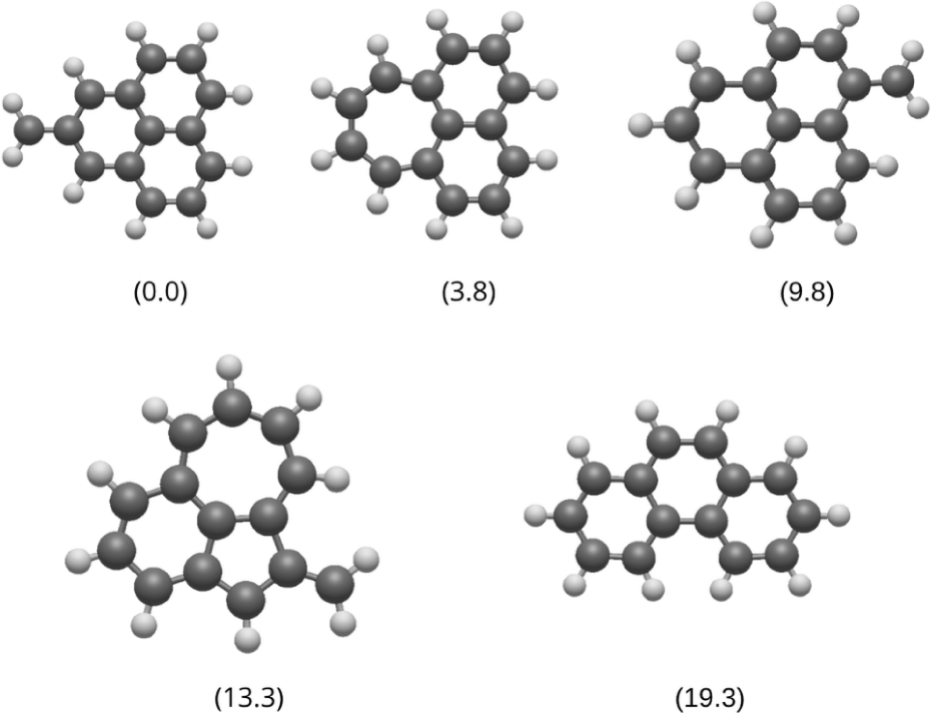
Low-energy structures obtained for phenanthrene dication by applying the automated reaction path search. The numbers in parentheses are the relative energies (Δ*E*) in kcal mol^–1^.

Despite containing a methylene group, the lowest-energy isomer (GM candidate) retains the structure of three six-membered fused rings characteristic of a phenalene core. This occurs because the condensation results from an increase in the number of C–C bonds shared between the rings, preserving the aromatic character of the system. This structure supports a resonance skeleton similar to that of neutral phenanthrene, significantly contributing to its stability. Moreover, the CH_2_ group facilitates better charge distribution in the dication (Figure S1), reducing internal repulsion and stabilizing the molecule. This result is in agreement with a previous study by Pereverzev et al.^28^ However, the researchers in that work employed a different theoretical method compared to the one used here. The fact that we identified the same global minimum structure for the phenanthrene dication as that of Pereverzev et al. demonstrates the validity of our AFIR-based method. Intriguingly, phenalene (C_13_H_10_) is the largest unsubstituted PAH detected to date in the ISM,^9^ indicating that its substituted counterparts, both neutral and ionized, might act as a milestone in the synthesis of more complex PAHs.

The second isomer exhibits an energy difference of 3.8 kcal mol^–1^ compared to that of the most stable candidate. Despite maintaining the same number of π electrons as neutral phenanthrene, the molecule has a seven-membered ring, which results in a charge distribution (Figure S2) distinct from that observed in neutral phenanthrene. Furthermore, the positive charge density generated by the loss of two electrons from the system is entirely contained within the ring atoms, unlike the most stable candidate, which has a CH_2_ group that allocates part of this charge. This charge distribution, anomalous compared to that of the lowest-energy candidate, explains the energy difference between the two structures. In the work of Pereverzev and coworkers, this structure was the third one in energy; the second isomer was the anthracene-like dication, which is missing from our list.^28^ These types of discrepancies are multicausal but could potentially arise from differences between the levels of theory used in our protocol, which lead to nonconverged structures in the mismatch between semiempirical and pure DFT methods. This is the reason why we use phenanthrene as a benchmark in this study.

The third most stable isomer has a structure very similar to that of the global minimum candidate. The energy difference of 9.8 kcal mol^–1^ can be attributed to the displacement of the CH_2_ group from the central ring to one of the peripheral ones, which significantly reduces the electric dipole moment and has effects on the charge distribution. The most stable candidate has a dipole moment of 3.99 D, while this third isomer has a reduced dipole moment of 2.54 D. When analyzing the dipole moment vector in both species (Figure S1), we observe that in both cases the vector points to the CH_2_ group. However, in the most stable candidate, the CH_2_ group is located in the central ring, causing the dipole moment vector to pass through the molecule center, promoting a more symmetric charge distribution and greater stability, and decreasing charge repulsion (Figure S1). In contrast, in the third isomer, the peripheral position of the CH_2_ group results in an asymmetric charge distribution, reducing the efficiency of stabilization. In this case, this isomer was not reported by Pereverzev and collaborators.^28^

The fourth isomer (lying 13.3 kcal mol^–1^ above the GM) exhibits characteristics similar to those observed in the second and third candidates. Its structure consists of three fused rings with unequal numbers of carbon atoms (5–6–7), creating an anomaly when compared to the neutral aromatic system and disrupting the electronic conjugation. Additionally, the sp^3^ hybridization of the carbon atom in the five-membered ring, which is directly bonded to the CH_2_ group, reduces the capacity of the CH_2_ carbon to stabilize the positive charge (Figure S3). These factors disturb the ideal charge distribution, contributing to the higher relative energy of this isomer.

The fifth low-energy isomer, lying 19.3 kcal mol^–1^ above the GM candidate, retains the same structure as that of neutral phenanthrene. The relative energy difference compared to the most stable candidate can be attributed to the inability of the neutral system, after losing two electrons, to efficiently maintain resonance. This induces isomerization into a condensed form in which the three rings share C–C bonds (see Figure S4 for bond lengths), favoring the stabilization of the dication. This situation is remarkably different when compared to the naphthalene dication, where the GM candidate has the naphthalene-like structure.^18^ This isomer was also reported by Pereverzev and coworkers.^28^ Linking this finding with the GM candidate, we conclude that ionic phenanthrene/anthracene-like structures favor isomerization into phenalinic structures, which might be one of the many reasons behind the nondetection of (neutral) phenanthrene or anthracene and the detection of phenalene (or their cyanide-substituted counterparts used as proxies in interstellar detection).^9^

With our protocol, we were able to localize important low-energy isomers and the GM of phenanthrene dication, in good agreement with a previous work, in which an exhaustive manual search was performed rather than an automated one.^28^ Now, we can discuss the low-energy phenanthridine dication obtained with the same validated protocol.

### 3.2 Low Energy Phenanthridine Dication Isomers

Following the identification of the global minimum candidates (Figure 3), we discuss the formation of these structures for phenanthridine^2+^. The energies of the structures were organized such that the lowest-energy candidate is represented as 0.0 kcal mol^–1^, in a manner similar to that of the phenanthrene^2+^ case.

**Figure 3:**
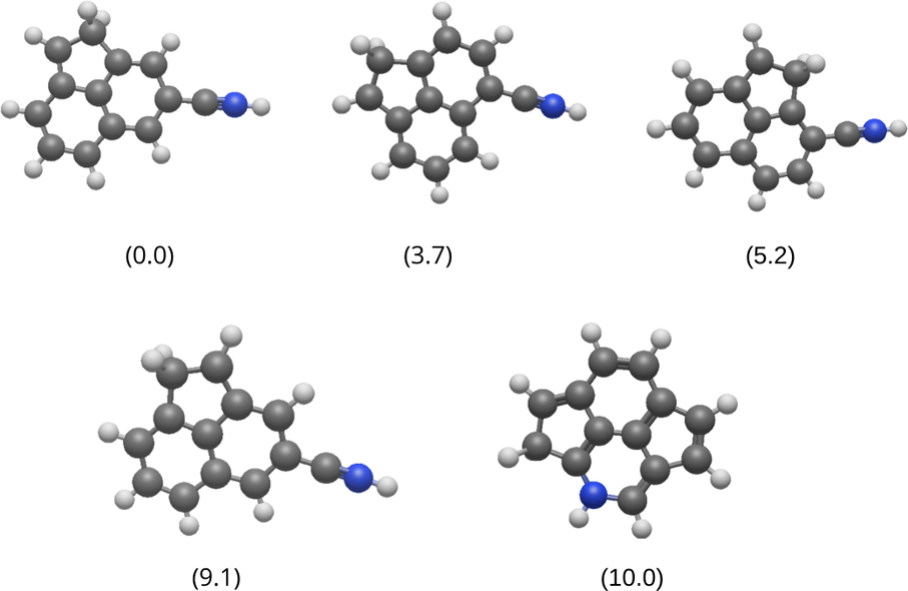
Low-energy structures obtained for phenanthridine dication by applying the automated reaction path search. The numbers in parentheses are the relative energies (Δ*E*) in kcal mol^–1^.

The first four most stable isomers of the phenanthridine dication share the same structural motif. They are hydrogenated aromatic nitriles composed of three fused rings: two with six carbons and one with five carbons forming an acenaphthene-like structure (similar to the biphenyl cation and dication^19^). This demonstrates that isomerization to a nitrile-like structure allows the positive charge density generated by the dication to be better stabilized by relocating the nitrogen outside of the ring system.

The most stable GM candidate has a C–N–H group attached to a carbon adjacent to two C–H groups. This position favors a more efficient charge distribution: the carbons bonded to hydrogen atoms acquire a partial positive charge (Figure S5), causing the carbon bonded to the nitrile to exhibit a significantly higher partial negative charge compared to the second isomer, where the C–N–H group is attached to a carbon that is also adjacent to another carbon shared by two rings. The greater polarization in the GM candidate generates a more intense dipole, providing greater electrostatic stability, which explains the energy difference of 3.7 kcal mol^–1^ and the dipole moment: 5.11 D for the most stable isomer and 3.77 D for the second one.

The third isomer (5.2 kcal mol^–1^ above the GM) shares the same feature as the second isomer: the C–N–H group is bonded to a carbon adjacent to one shared by two rings. However, in this case, the neighboring ring is a five-membered one that includes an sp^3^ carbon. Although this sp^3^ carbon does not directly contribute to the delocalization effects, its proximity to the boundary carbon adjacent to the C–N–H group alters the local bond lengths and angles when compared to the other two more stable isomers (Figure S6), destabilizing the π system and charge localization (Figure S5).

The fourth isomer (9.1 kcal mol^–1^) is very similar to the most stable candidate. The sp^2^ carbon of the CH_2_ group is positioned adjacent to a carbon common to the two rings. The partially negative charge of the carbon in the CH_2_ group (Figure S5) and the already discussed differences in bond lengths and angles affect the electrostatic stability of the isomer and its delocalization effects. This difference in charge distribution also shifts the dipole moment vector, which, despite being larger in magnitude (7.81 D), points farther away from the C–N–H group compared to the more stable isomer (Figure S5).

The least stable candidate (10.0 kcal mol^–1^) is the only one among the five that does not exhibit the three-ring structure with a C–N–H group. This isomer is the only one that retains the nitrogen within the ring, similar to neutral phenanthridine. Analogous to the dication of phenanthrene, this structure isomerizes into a more condensed system, with more carbon atoms shared among the rings. However, this increased condensation is still not as effective at managing the positive charge density as the rearrangement into a nitrile-like structure (Figure S7). This reinforces the idea that isomerization to an aromatic nitrile more efficiently stabilizes the positive charge density of the dication, highlighting a clear structural preference.

Interestingly, the energy window of the low-lying isomers of PANHs^2+^ is narrower than that in the case of PAHs^2+^. This points to the prevalence of nitrile groups as charge acceptors, not only in dications but more generally in cationic nitriles, as illustrated, for example, in studies of ionized and protonated benzonitrile.^80,81^ Similar to phenanthrene, the chemical skeletons of the four lowest isomers of phenanthridine^2+^ correspond to (protonated) cyano-acenaphthylene, a cyano derivative of PAHs already detected in the ISM,^82^ in contrast with neutral phenanthridine. The fact that our ionic structures follow trends observed in astronomical detections is certainly intriguing. The identification of one isomer of a PAH over another in space likely depends on many factors, involving not only isomerization pathways but also formation and destruction routes. Our calculations, however, suggest possible new avenues to rationalize the still unresolved chemical complexity of PAHs in the ISM, where seemingly more stable isomers (or their cyano derivatives) are not always those detected, although in some cases they are.^40,42^

### 3.3 Simulated Infrared Spectra

#### 3.3.1 Phenanthrene^2+^: First Isomer

All simulated IR spectra are listed in Figure 4. The IR spectrum of the lowest-energy isomer, the candidate for GM, shows its most intense peak at 1640 cm^–1^. This peak is characterized by the vibrational modes of stretching (ν C=C) and in-plane bending C–C–H (δ C–C–H) that involve all three rings. The second most intense peak occurs at 1210 cm^–1^, corresponding to the in-plane bending modes of C–C–H (δ C–C–H). In the lower intensity regions, the peak at 1396 cm^–1^ exhibits the normal modes of in-plane bending C–C–H (δ C–C–H) bonds and the scissor mode of the CH_2_ group. At 1600 cm^–1^, the vibrational modes include an in-plane bending mode of C–C–H (δ C–C–H) and an accentuated scissor mode of the CH_2_ (δ H–C–H) group. At 1530 cm^–1^, the normal modes include stretching (ν C=C) and in-plane C–C–H bendig mode (δ C–C–H). The difference between the 1640 cm^–1^ and 1530 cm^–1^ modes lies in the shape of the stretching mode. While at 1640 cm^–1^ a symmetrical stretching of the ring occurs, at 1504 cm^–1^ there is asymmetrical stretching.

**Figure 4:**
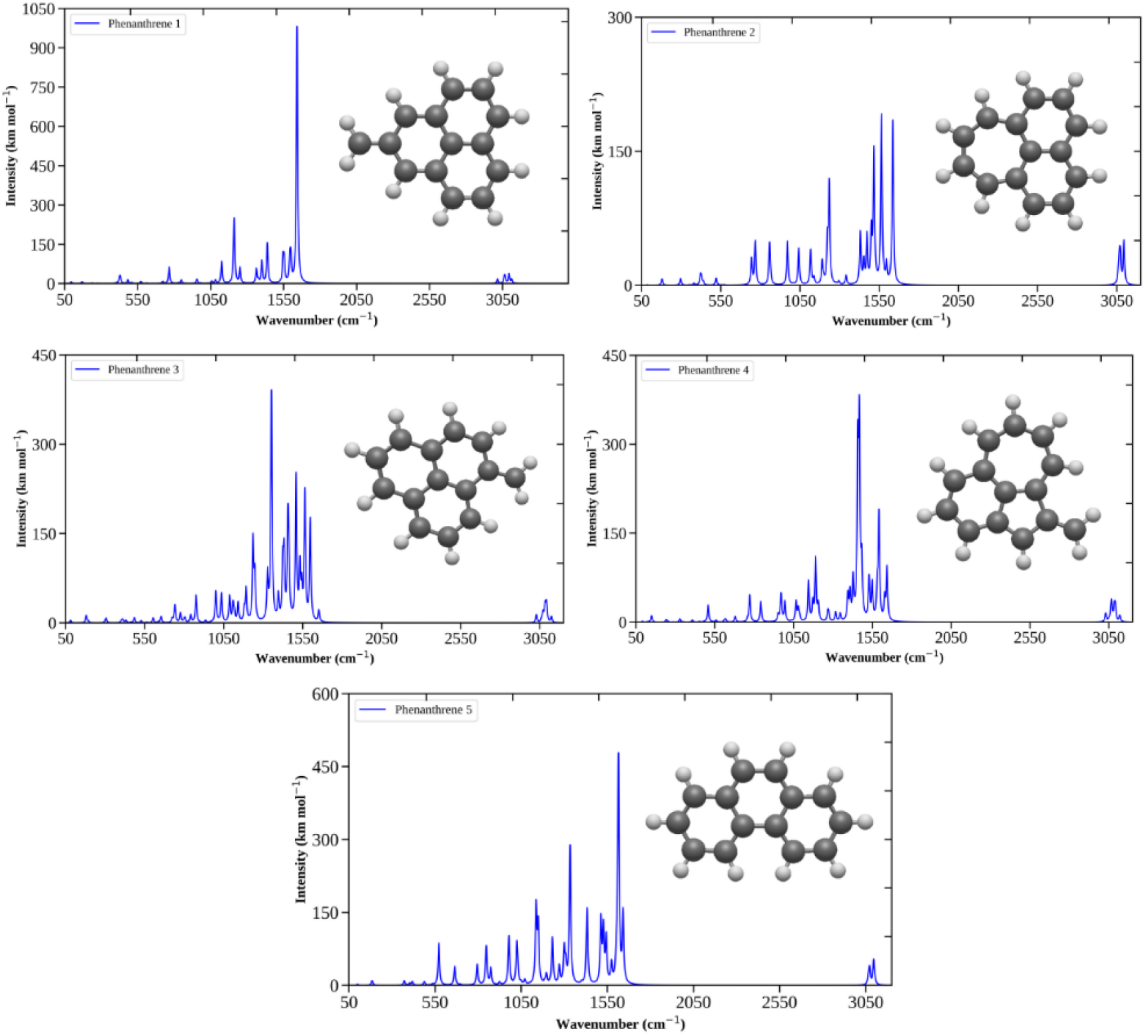
Simulated infrared spectra of phenanthrene low-energy isomers.

#### 3.3.2 Phenanthrene^2+^: Second Isomer

The most intense peak in the IR spectrum for this isomer is in the range of 1560 cm^–1^ and is characterized by the in-plane bending mode of C–C–H (δ C–C–H) with a greater predominance of the mode in C–C–H bonds exclusive to the 7-membered ring. The second most intense peak, 1630 cm^–1^, is attributed to the normal mode of ring (ν C=C). The third one, 1500 cm^–1^, occurs due to the in-plane bending modes of C–C–H (δ C–C–H). The mode occurs in the three rings with the same amplitude.

The peak at 1224 cm^–1^, like the most intense one (1553 cm^–1^), corresponds to the normal modes of C–C–H in-plane bending (δ C–C–H). At 1224 cm^–1^, however, the normal mode occurs predominantly in the peripheral carbons of 6-membered rings. Finally, the peak at 3160 cm^–1^ presents the stretching of C–H (ν C–H) bonds in the 6-membered rings.

#### 3.3.3 Phenanthrene^2+^: Third Isomer

The two most intense peaks in the IR spectrum for the third isomer are located around 1350 cm^–1^ and 1500 cm^–1^. These two peaks exhibit the same normal vibrational modes: stretching (ν C=C) and an in-plane bending mode (δ C–C–H). The difference between them lies in how these vibrational modes occur. At 1350 cm^–1^, one of the C–H bonds in CH_2_ performs the C–C–H (δ C–C–H) bending mode, which is the most intense mode in this region. At 1500 cm^–1^, the C–C–H (δ C–C–H) does not have the contribution of an H from the CH_2_ group. The most intense normal mode in this region is the stretching (ν C=C). At 1560 cm^–1^, the normal mode is characterized by the C–C stretching (ν C–C) between the C atom ring and the C atom of the CH_2_ group. The other mode at 1560 cm^–1^ is the CH_2_ scissor (δ H–C–H). The peak at 1456 cm^–1^ corresponds to stretching (ν C=C) and the in-plane bending mode of C–C–H bonds (δ C–C–H). Here, the two modes have equivalent amplitude. At 1230 cm^–1^, there is a contribution from the bending modes of the C–C–H bonds (δ C–C–H).

#### 3.3.4 Phenanthrene^2+^: Fourth Isomer

The most intense peak in the IR spectrum for this isomer occurs at 1460 cm^–1^ and is represented by the in-plane bending mode of C–C–H (δ C–C–H) bonds and CH_2_ group scissors (δ H–C–H). The second peak occurs at 1600 cm^–1^ and it is represented by in-plane bending C–C–H (δ C–C–H) and stretching (ν C=C).

The other bands with lower intensity are 1640 cm^–1^, 1190 cm^–1^, and 1144 cm^–1^. At 1640 cm^–1^ occurs C–C stretching (ν C–C) between C atom of the 5-membered ring and the C atom of the CH_2_ group. The other normal mode present at 1640 cm^–1^ is the CH_2_ group scissor. The 1190 cm^–1^ is characterized by an intense in-plane bending C–C–H (δ C–C–H) bond in the 5-membered ring. At 1144 cm^–1^ occurs the normal mode of in-plane bending (δ C–C–H) of C–C–H bonds of 5- and 6-membered rings. The other mode at 1144 cm^–1^ is the stretching (ν C=C) of the 7-membered ring.

#### 3.3.5 Phenanthrene^2+^: Fifth Isomer

The isomer with higher energy for phenanthrene^2+^ is the only one that maintains a phenanthrene-like structure (neutral state). The most intense peak in its IR spectrum occurs at 1610 cm^–1^, stretching (ν C=C) of the central ring. The second most intense peak, at 1330 cm^–1^, exhibits the same vibrational mode as the bending mode at 1610 cm^–1^ (ν C=C), but only in the lateral rings.

The peak at 1135 cm^–1^ is composed of the in-plane bending C–C–H (δ C–C–H) in the exclusive bonds of side rings. The peaks at 1417 cm^–1^ exhibit stretching (ν C=C) at the central ring and in-plane bending of C–C–H (δ C–C–H) bonds of lateral rings. The peak at 1512 cm^–1^ shows the stretching in the three rings (ν C=C).

#### 3.3.6 Phenanthridine^2+^: First Isomer

All simulated IR spectra are listed in Figure 5. The infrared spectrum of the lowest-energy isomer (GM candidate) features its most intense peak at 3483 cm^–1^, attributed to stretching of the N–H bond (ν N–H). The second one occurs at 2345 cm^–1^ and is due to the stretching of the C–N bond (ν C–N).

**Figure 5:**
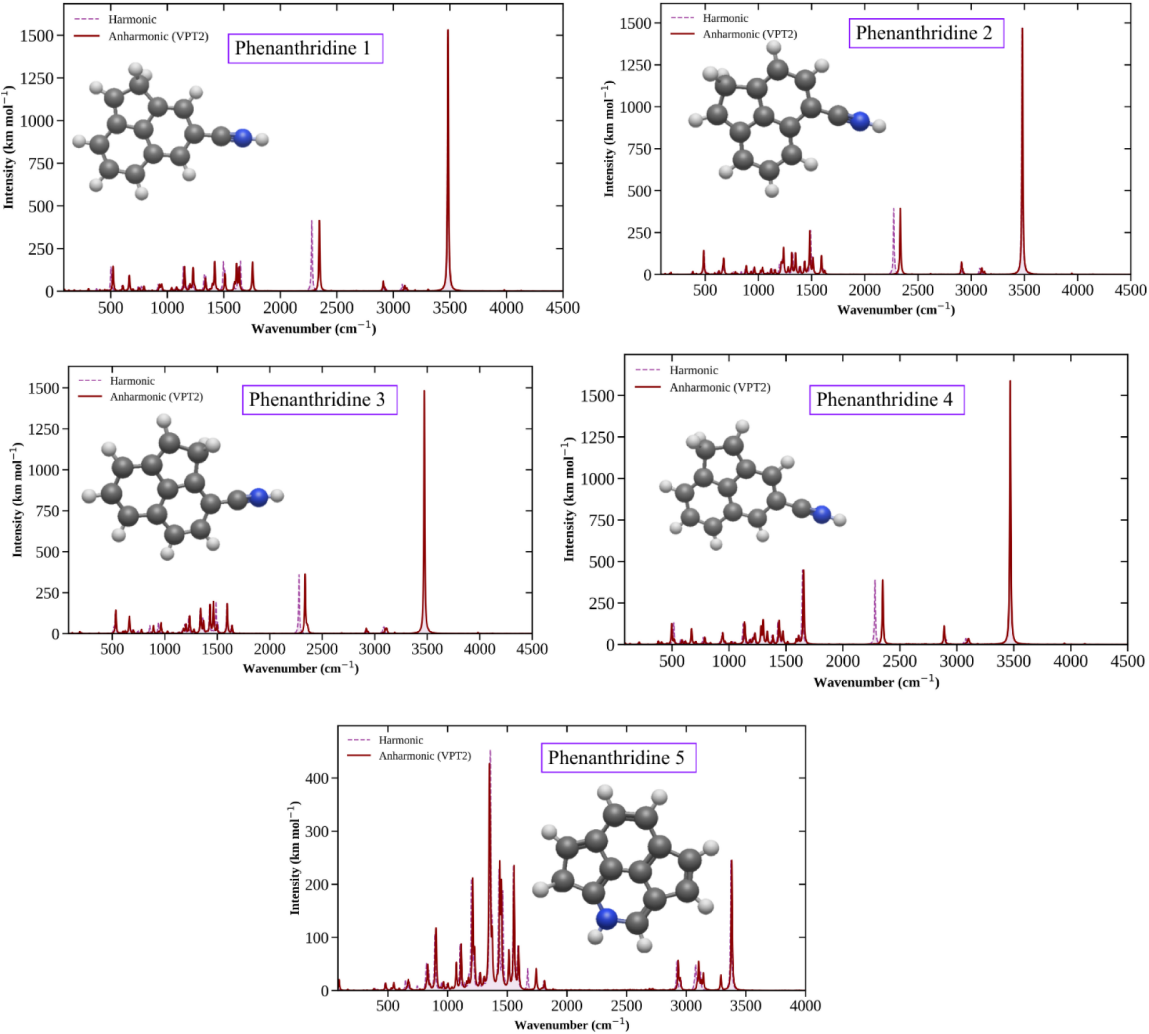
Simulated infrared spectra of phenanthridine low-energy isomers. Lines in purple: harmonic frequencies were obtained with two scaling factors. Lines in red: anharmonic frequencies were obtained with VPT2.

The peak at 1754 cm^–1^ is characterized by the vibrational modes of stretching (ν C=C), with contributions from the in-plane bending mode of C–C–H (δ C–C–H). Another intense peak occurs at 1613 cm^–1^ with similar stretching vibrational modes. The difference between these peaks lies essentially in the regions where vibrational modes occur. At 1754 cm^–1^, the stretching and in-plane bends occur with greater amplitude in the ring where the C–N–H group is attached. In contrast, at 1613 cm^–1^, the vibrational modes occur in the other rings, with the addition of the scissoring mode of the CH_2_ (δ H–C–H) group. Finally, the band at 1152 cm^–1^ corresponds to the stretching (ν C=C), combined with in-plane bendings of C–C–H bonds (δ C–C–H) and CH_2_ wagging (γ H–C–H).

#### 3.3.7 Phenanthridine^2+^: Second Isomer

Similarly to the main GM candidate and the other structures whose isomerization resulted in a nitrile, the most intense peak of this structure occurs at 3481 cm^–1^, in the region of stretching of the N–H bond (ν N–H). The second most intense band appears at 2333 cm^–1^, attributed to the stretching of the C–N bond (ν C–N).

The peak at 1483 cm^–1^ is characterized by stretching (ν C=C), with contributions from the in-plane bending mode of C–C–H (δ C–C–H) and CH_2_ scissoring (δ H–C–H). At 486 cm^–1^, the out of plane bending N–H (γN–H) bond is observed, a vibrational mode that exclusively appears in this isomer among the most intense bands. Finally, the band at 1349 cm^–1^ is attributed to the stretching (ν C=C), with contributions from the in-plane bending mode of C–C–H (δ C–C–H) and CH_2_ scissoring (δ H–C–H). Unlike the mode at 1483 cm^–1^, which had the strongest stretching mode in the bonds of the five-membered ring, the mode at 1349 cm^–1^ exhibits the strongest stretching intensity in the six-membered rings. Furthermore, the scissor mode of the CH_2_ group is the most intense at 1349 cm^–1^.

#### 3.3.8 Phenanthridine^2+^: Third Isomer

The IR spectrum of this isomer presents its most intense peak at 3472 cm^–1^, which is characterized by the stretching of the N–H bond (ν N–H). The second most intense band occurs at 2336 cm^–1^, attributed to the stretching modes of the C–N bonds (ν N–H).

Concerning the lower intensity bands, in the peak at 1463 cm^–1^, where observed stretching (ν C=C) mode, in-plane bending of the C–C–H bonds (δ C–C–H) and CH_2_ scissors (δ H–C–H) are observed. The mode at 1595 cm^–1^ is similar to the one at 1463 cm^–1^. The difference lies in the CH_2_ group and localization of the stretching intensities. At 1595 cm^–1^, the CH_2_ group performs a slight wagging (γ H–C–H). At 1463 cm^–1^, the stretching (ν C=C) was strongest at the individual C=C bonds of the 5- and 6-membered rings. At 1595 cm^–1^, on the other hand, the stretching (ν C=C) is strongest at the C=C bonds shared by the 5- and 6-membered rings. The mode at 1431 cm^–1^ is similar to the mode at 1463 cm^–1^. The difference is only in the location of the stretching intensities. At 1463 cm^–1^, the stretching (ν C=C) is strongest at the individual C=C bonds of the 5- and 6-membered rings. At 1431 cm^–1^, on the other hand, the stretching (ν C=C) is strongest at the individual C=C bonds of the 6-membered rings.

#### 3.3.9 Phenanthridine^2+^: Fourth Isomer

The IR spectrum of this structure, a constitutional isomer of the most stable one (the change occurs only in the position of the CH_2_ group in the 5-membered ring), shows its most intense peak in the region of 3468 cm^–1^, corresponding to the stretching of the N–H (ν N–H) bond. Following this, the stretching (ν C=C) and in-plane bend mode of the C–C–H bonds (δ C–C–H), with a peak at 1612 cm^–1^, represents the second most intense peak, and it corresponds to the stretching mode (ν C=C).

The peak at 2349 cm^–1^ is attributed to the vibrational mode of the stretching of the C–N bond (ν C–N). The peak at 1299 cm^–1^ corresponds to the CH_2_ scissor mode (δ H–C–H) and in-plane bending of C–C–H (δ C–C–H). Finally, the last significant peak, observed at 497 cm^–1^, is characterized by the out-of-plane bending of N–H (γ N–H).

#### 3.3.10 Phenanthridine^2+^: Fifth Isomer

The highest-energy candidate is the only one, among the five candidates for the global minimum of phenanthridine dication, that does not undergo isomerization to an aromatic nitrile. For this reason, it is the only one whose most intense peak is not the band in the region of N–H stretching (ν N–H), at 3382 cm^–1^, which instead corresponds to the second most intense band in its IR spectrum. Its most intense band occurs at 1350 cm^–1^, associated with the in-plane bending mode of C–C–H (δ C–C–H) and the scissor mode of CH_2_ (δ H–C–H). At 1556 cm^–1^, the stretching (ν C=C), combined with the in-plane bend modes of the C–N–H (δ C–N–H) and C–C–H (δ C–C–H) bonds, is observed.

The peak at 1436 cm^–1^ is characterized by the stretching (ν C=C) and the in-plane bend mode of C–C–H (δ C–C–H) bonds. Finally, the band at 1210 cm^–1^ involves the stretching (ν C=C) and an intense bending mode of the C–C–H bond on the carbon adjacent to the CH_2_ group.

## 4 Discussion

It is known that multiple ionizations can trigger several isomerization reactions following internal energy redistribution. For instance, small aromatics such as benzene and toluene undergo molecular rearrangement after the double ionization process.^23,27^ As a consequence, all geometry-dependent properties and spectra will be modified. In the case of benzene dication, the IR spectra are highly dependent on the isomer’s molecular geometry.^22,24,25,58^ For the nitrogen-bearing aromatic molecules such as aniline, isomerization reactions were also reported after the multiple ionization process.^57^ Similar results were obtained for naphthalene,^18^ biphenyl,^19^ and phenanthrene^28^ where the IR spectra exhibit differences that depend on the geometry or charge state of the isomers.

In this work, we applied a GM search for phenanthrene (as a benchmark case) and phenanthridine at the dication charge state. We found similar results reported by Pereverzev and coworkers,^28^ demonstrating that automated methods are efficient in the search for low-energy isomers. Phenanthrene isomerization reactions occur after the ionization process, with all isomers having the most intense bands between 1400 cm^–1^ and 1600–1400 cm^–1^ related to C–C–H bending and C–C stretching modes. As expected, for cations and dications, the most intense bands are not in the same region of the spectrum when compared to the neutral state.^18,19,28,33,83,84^ Also, as suggested by Pereverzev and coworkers,^28^ low-energy isomers can contribute to the IR spectrum of the dication species.

Concerning phenanthridine dication, isomerization also occurs with the four lowest-energy isomers corresponding to positional isomers of the same acenaphthylenic protonated nitrile, i.e., the formation of the CNH group bonded to the core rings. A very different IR spectrum profile was obtained. Unlike the spectrum of neutral phenanthridine, whose most intense bands are in the region below 1000 cm^–1^,^85^ the most intense bands are present around 2300 cm^–1^ due to C–N stretching and in the range of 3500 cm^–1^ (N–H stretching). Further, the IR spectrum of the phenanthridine cation is also different, with the main bands around 700 cm^–1^ and 1200 cm^–1^.^30^ The fifth isomer has an IR spectrum similar to phenanthrene low-energy isomers with the main bands between 1400 cm^–1^ and 1500 cm^–1^. Although there are bands around 6.2 μm (1610 cm^–1^), these bands are not very intense, indicating that these dications are unlikely to contribute to the AIBs, in contrast to what some authors argue for neutral and cationic PANHs.^30,44,49,56^

The data obtained with scaling factors (applied to harmonic frequencies) are very similar to the anharmonic (VPT2) results. The joint analysis of the intrinsic ν_anharm_/ω_harm_ ratios (Tables S11–S15) and the visual inspection of the spectra (Figures 5, S11, S13–S15) shows that the applied scaling factors are valid. Notably, the most intense peaks in the 3500 cm^–1^ region exhibit excellent agreement, with deviations smaller than 10 cm^–1^. In the intermediate region (around 2500 cm^–1^), the difference between harmonic and anharmonic frequencies never exceeds 60 cm^–1^. The sacling factors used in this work agree well within the calculated standard deviation for all isomers, corroborating the accuracy of the discussed data. In the fingerprint region (500–2000 cm^–1^), the application of the dual scaling factor demonstrated excellent agreement for the vast majority of spectroscopically relevant vibrational modes. The sole significant discrepancy was observed in the most stable isomer (Isomer 1), concerning an isolated mode around 1750 cm^–1^, which exhibited a deviation of 107 cm^–1^ relative to the anharmonic value. However, disregarding this specific exception, the accuracy for all other significant peaks across the five isomers remained robust, with deviations consistently below 20 cm^–1^.

For the five isomers, overtone and combination bands are observed in the regions 1800–2200 cm^–1^ and 2800–3300 cm^–1^. These bands are also present in the 3600–5000 cm^–1^ range for the first four isomers. However, this region has no bands for the least stable phenanthridine dication isomer. These features can be observed in detail in Figures S13–S15. For the first four isomers, the most intense combination bands appear in the 3800–4200 cm^–1^ range, with intensities around 7 km mol^–1^. Opposite, for the fifth isomer, the most significant contribution from these bands occurs between 1850 and 1900 cm^–1^, with intensities not exceeding 3 km^–1^. These results indicate that the contributions from overtones and combination bands are negligible for the detection of those species.

Comparing dipole moments, low-energy isomers of phenanthridine dications exhibit the higher ones, from 3.80 to 7.81 D, which have the same magnitude or are even bigger when compared to cyanopyrene^40^ and cyanocoronene,^42^ which were detected in the TMC-1. A high dipole moment encourages the search for these species via radio astronomy.

We would like to highlight the serendipitous finding that both the global minimum and the low-lying isomers of phenanthrene and phenanthridine dications share a structural skeleton with interstellar molecules that have been positively identified, namely phenalene^9^ and cyano-acenaphthylene,^82^ in contrast to phenanthrene and phenanthridine themselves, which remain undetected. It is important to note that cyanonaphthalene and phenanthridine do not share the same molecular formula (C_13_H_8_N vs C_13_H_9_N), but they do exhibit a similar structural backbone. This observation motivates us to further investigate the role of geometric motifs in the PAH complexity ladder with the goal of establishing correlations between ionized, doubly ionized, and protonated (N-)­PAHs and their neutral counterparts. Such correlations may provide subtle indicators of the energetic processing driven by ionizing radiation in the formation of interstellar PAHs.

Regarding the formation of these doubly charged species, both vertical and adiabatic ionization potentials were calculated considering the transitions from neutral phenanthrene to the phenanthrene dication and from neutral phenanthridine to the phenanthridine dication, as depicted in Figures S14 and S15 of SI, respectively. The calculations revealed ionization values of 19 eV (adiabatic) and 20 eV (vertical) for phenanthrene, while for phenanthridine, the values are 19 eV (adiabatic) and 21 eV (vertical). The presence of singly ionized PAHs in the ISM is suggested by the relatively low observed intensity ratios between the 11.3 and 7.7 μm bands, which are attributed to neutral and ionized PAHs, respectively. This is particularly true in star-forming regions in our galaxy and in external galaxies. In harsher environments (e.g., around actively accreting black holes at the centers of active galaxies, AGN) with more intense far-UV and X-ray radiation, doubly ionized PAHs, similar to the dications studied here, might be present. Moreover, in regions subject to high cosmic ray ionization rates, such as around supernova remnants or AGN, He^2+^ and He^+^, present in the cosmic ray spectrum, as well as $H_{3}^{+}$ , formed after the ionization of H_2_ in the ISM, can lead to the formation of dication PAHs through the direct ionization, or protonation + ionization, of existing neutral PAHs. We emphasize, however, the speculative nature of this result, which will require a more extensive investigation in future studies.

## 5 Conclusion

In this work, we successfully applied an automated search for GM and low-energy isomer structures through the SC-AFIR method. Our benchmark on phenanthrene dication confirmed the results obtained by Pereverzev and coworkers,^28^ where the GM has three fused six-membered rings of a phenalene core bonded to a methylene group with a high dipole moment of 3.99 D. It is worth mentioning that the GM structure is quite different from benzene,^23,24^ naphthalene,^18^ and biphenyl^19^ GM geometries. The high relative stability can be explained by the electrostatic effect, that is, by the homogeneous distribution of charges. The low-energy isomer IR spectra are similar to those of PAH cations, and the intense bands are located around 1500 cm^–1^ due to C–C–H bending and C–C stretching modes.

Low-energy isomers of phenanthridine dication have a very different molecular geometry than the neutral counterpart, indicating a huge isomerization after the double ionization process. Remarkably, the first four low-energy structures are protonated nitriles with a CNH group bonded to three fused rings (acenaphthylene-like structure, similar to the biphenyl case^19^). Once again, charge distribution seems to be the main stabilization factor. Computed dipole moments are very high, from 3.80 to 7.81 D, which may encourage the detection of these species, since these values are compatible or even higher when compared to the dipole moments of the recently detected cyanopyrene^40^ and cyanocoronene.^42^ Besides, we find that double ionization promotes a series of isomerizations that are compatible with the molecular structures of phenalene and acenaphthylene found in the ISM.^9,82^

Infrared spectra of low-energy isomers of phenanthridine dication have a different profile when compared to those of phenanthrene and PAH cations. The spectral signatures of CNH are present, i.e., intense bands between 2000 cm^–1^ and 2500 cm^–1^ due to the C–N stretching mode. Also, above 3500 cm^–1^, intense bands are present due to N–H stretching. Less intense bands are present around 6.2 μm (1610 cm^–1^), indicating that these species are unlikely to contribute to the AIBs, different from what was proposed for PANHs at neutral and cation states.^30,44,49,56^

The comparison between the harmonic spectra, treated with dual scaling factors, and the anharmonic spectra obtained via VPT2 shows that the dual scaling approach is sufficiently robust for the infrared spectral simulations of these isomers, as evidenced by both visual comparisons (spectral plots) and quantitative analysis (frequency ratios). Indeed, while there are spectral regions where the harmonic approximation predicts no features (containing only overtones and combination bands), these contributions are not very relevant to the final spectral profile due to the low intensities. Consequently, these features are considered negligible, and the use of scaling factors results are accurate enough for primary detection purposes.

## Supplementary Material



## Notes

Infrared band assignment, additional molecular structures, simulated infrared spectra, ionization potentials, and XYZ structures (PDF)
