# Supplementary material for: Double Ionization of Polycyclic Aromatic Nitrogen Heterocycles: Simulations on Phenanthridine
Source: ACS Earth Space Chem. 2026 Jan 30;10(2):370–81. doi: 10.1021/acsearthspacechem.5c00240 (PMC12928202; doi:10.1021/acsearthspacechem.5c00240)
Supplement: Supplementary file 1 [file sp5c00240_si_001.pdf]

Supporting Information: Double Ionization of Polycyclic Aromatic Nitrogen Heterocycles: Simulations on Phenanthridine

Jorge H. C. Basilio,[†] Germán Molpeceres,[*,‡] Wania Wolff,[†] Miguel Pereira-Santaella,[‡] and Ricardo R. Oliveira[*,¶]

†*Physics Institute, Federal University of Rio de Janeiro, Rio de Janeiro, RJ 21941-909, Brazil.*

‡*Departamento de Astrofísica Molecular, Instituto de Física Fundamental (IFF-CSIC), C/Serrano 121, 28006 Madrid, Spain.*

¶*Chemistry Institute, Federal University of Rio de Janeiro, Rio de Janeiro, Brazil - 21941-909*

E-mail: german.molpeceres@iff.csic.es; rrodrigues.iq@gmail.com

Contents

1 Infrared band assignment **S-3**

2 Molecular structures **S-6**

3 Simulated infrared spectra **S-11**

4 Ionization Potentials **S-17**

5 Cartesian Coordinates of Phenanthrene dications **S-18**

6 Cartesian Coordinates of Phenanthrene dications **S-34**

7 Fundamental transitions - harmonic vs anharmonic calculations **S-49**

1 Infrared band assignment

Table S1: data of most intensities bands in phenantrene-1^{2+}

**Wavenumber (cm^{-1})**	**Intensity (km mol^{-1})**	**Group**	**Mode**
1640	990	C=C/C_2-H	ν C=C/ δC-C-H.
1210	250	C-H_2/ C_2-H	δC-C-H/ δ H-C-H
1396	155	C_2H/C-H_2	δ C-C-H./ δ H-C-H .
1600	136	C_2H/C-H_2	δ H-C-H .
1530	119	C=C/C_2-H	ν C=C/ δ C-C-H

Table S2: data of most intensities bands in phenantrene-2^{2+}

**Wavenumber (cm^{-1})**	**Intensity (km mol^{-1})**	**Group**	**Mode**
1560	190	C_2-H	δ C-C-H
1630	184	C=C	ν C=C
1500	154	C_2-H	δ C-C-H
1224	118	C_2-H	δ C-C-H
3160	50	C-H	ν C-H

Table S3: data of most intensities bands in phenantrene-3^{2+}

Wavenumber (cm^{-1})	Intensity (km mol^{-1})	Group	Mode
1350	390	C=C/C_2-H	(ν C=C)/(δ C-C-H)
1500	251	C=C/C_2-H	(ν C=C)/(δ C-C-H)
1560	226	C-C/C-H_2	ν C-C/δ H-C-H
1456	118	C=C/C_2-H	(ν C=C)/ (δ C-C-H)
1230	150	C_2-H	(δ C-C-H)

Table S4: data of most intensities bands in phenantrene-4^{2+}

Wavenumber (cm^{-1})	Intensity (km mol^{-1})	Group	Mode
1460	380	C_2-H/C-H_2/	(δ C-C-H)/(δ H-C-H)
1600	190	C=C/C_2-H	(δ C-C-H)/(ν C=C)
1640	109	C-C/C-H_2/	(ν C-C)/(δ H-C-H)
1190	92	C_2-H	(δ C-C-H)
1140	67	C=C/C_2-H/	(ν C=C)/ (δ C-C-H)

Table S5: data of most intensities bands in phenantrene-5^{2+}

Wavenumber (cm^{-1})	Intensity (km mol^{-1})	Group	Mode
1610	480	C=C	(ν C=C)
1330	286	C=C	(ν C=C)
1135	172	C_2-H	(δ C-C-H)
1417	159	C=C/C_2-H	(ν C=C) / (δ C-C-H)
1512	143	C=C	(ν C=C)

Table S6: data of most intensities bands in phenantridine-1^{2+}

Wavenumber (cm^{-1})	Intensity (km mol^{-1})	Group	Mode
3483	1527	N-H	(ν N-H)
2345	413	C-N	(ν C-N)
1754	171	C=C/C_2-H/	(ν C=C)/ (δ C-C-H)
1613	171	C=C/C_2-H/	(ν C=C)/ (δ C-C-H)
1152	138	C=C/C_2-H/C-H_2	(ν C=C)/(δ C-C-H)/ (γ H-C-H)

Table S7: data of most intensities bands in phenantridine-2^{2+}

**Wavenumber (cm^{-1})**	**Intensity (km mol^{-1})**	**Group**	**Mode**
3481	1527	N-H	(ν N-H)
2333	388	C-N	(ν C-N)
1483	256	C=C/C_2-H/C-H_2	(ν C=C)/(δ C-C-H)/ (δ H-C-H)
486	138	N-H	(γ N-H)
1349	124	C=C/C_2-H/C-H_2	(ν C=C)/(δ C-C-H)/ (δ H-C-H)

Table S8: data of most intensities bands in phenantridine-3^{2+}

**Wavenumber (cm^{-1})**	**Intensity (km mol^{-1})**	**Group**	**Mode**
3472	1474	N-H	(ν N-H)
2336	358	C-N	(ν C-N)
1463	195	C=C/C_2-H/C-H_2	(ν C=C)/(δ C-C-H)/ (δ H-C-H)
1595	174	C=C/C_2-H/C-H_2	(ν C=C)/(δ C-C-H)/ (δ H-C-H)
1431	174	C=C	(ν C=C)

Table S9: data of most intensities bands in phenantridine-4^{2+}

**Wavenumber (cm^{-1})**	**Intensity (km mol^{-1})**	**Group**	**Mode**
3468	1574	N-H	(ν N-H)
1612	174	C=C	(ν C=C)
2349	358	C-N	(ν C-N)
1300	143	C_2-H/C-H_2/	(δ C-C-H)/(δ H-C-H)
497	124	N-H	(γ N-H)

Table S10: data of most intensities bands in phenantridine-5^{2+}

**Wavenumber (cm^{-1})**	**Intensity (km mol^{-1})**	**Group**	**Mode**
1350	448	C_2-H/C-H_2/	(δ C-C-H)/(δ H-C-H)
3382	242	N-H	(ν N-H)
1556	234	C=C/C_2-H/C-H_2	(ν C=C)/(δ C-C-H)/ (δ C-N-H)
1436	224	C=C/C_2-H/	(ν C=C)/ (δ C-C-H)
1210	208	C=C/C_2-H/	(ν C=C)/ (δ C-C-H)

2 Molecular structures

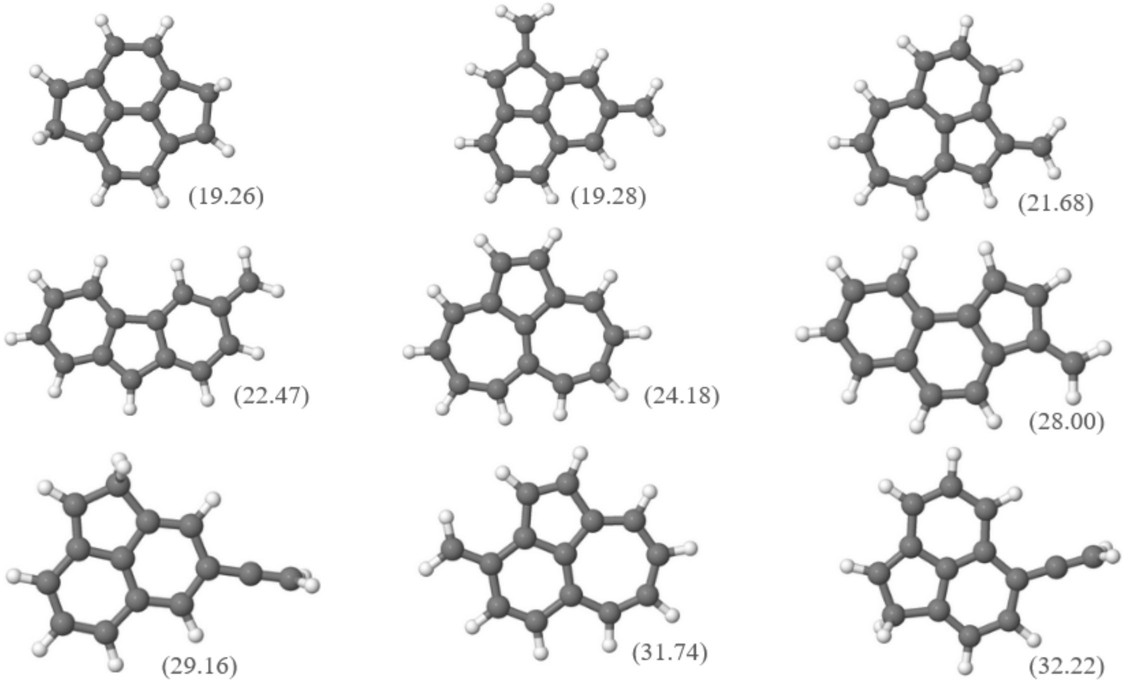

Figure S1: Other structures obtained for phenantrene dication applying the automated reaction path search. The numbers in parenthesis are the relative energies (ΔE) in kcal mol^{-1}.

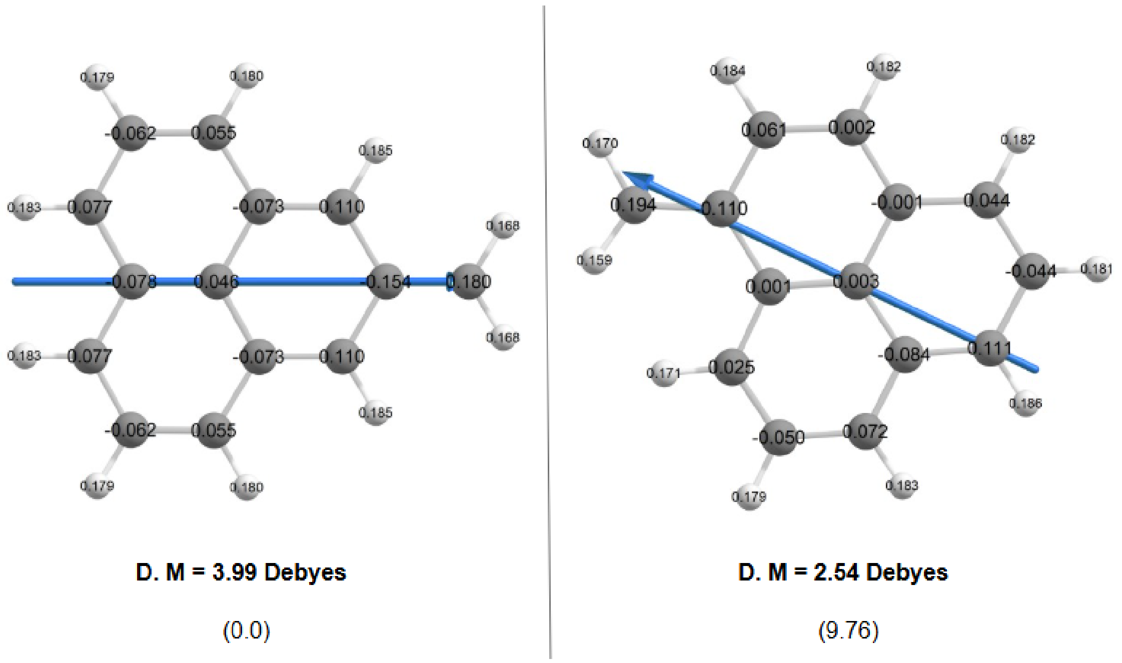

Figure S2: Dipole moments and charge distribution of the most stable and the third most stable G.M candidates for phenanthrene^{2+}. The numbers in parentheses are the relative energies (ΔE) in kcal mol^{-1}.

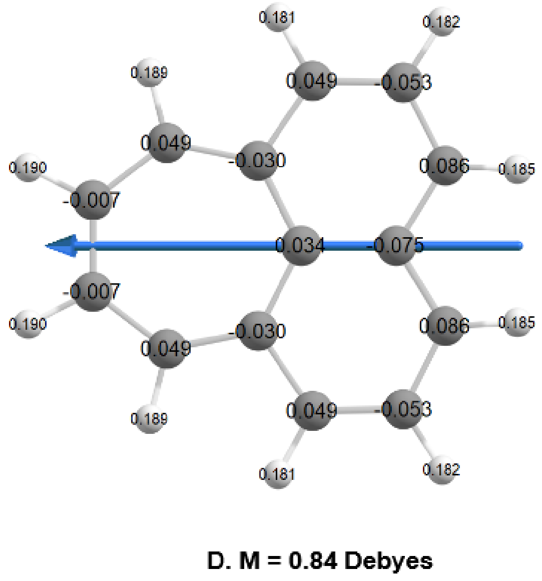

Figure S3: Dipole moment vector and charge distribution of the second most stable G.M candidate for phenanthrene^{2+}.

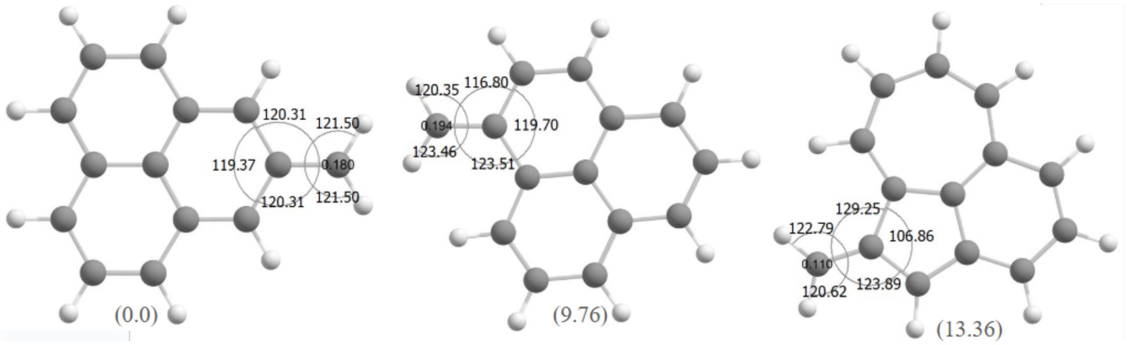

Figure S4: Bond angles involving the CH_2 carbon and its vicinal carbon, along with the partial charge of the CH_2 carbon, for phenanthrene^{2+} isomers. The numbers in parentheses indicate the relative energies (ΔE) in kcal mol^{-1}.

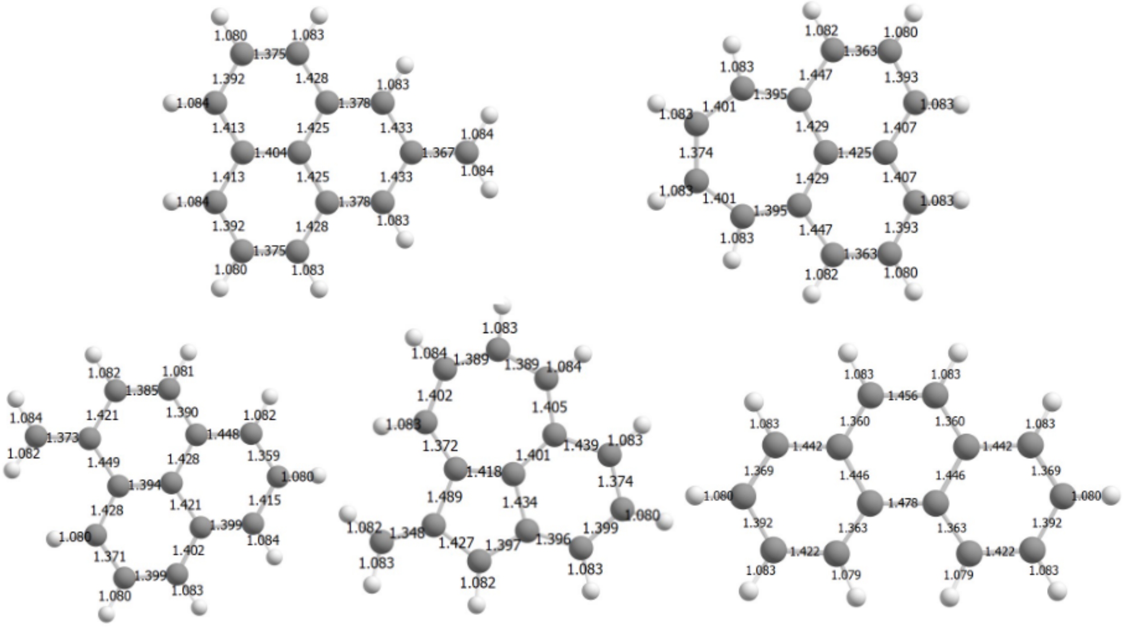

Figure S5: Bond lengths for all phenanthrene^{2+} G.M candidates.

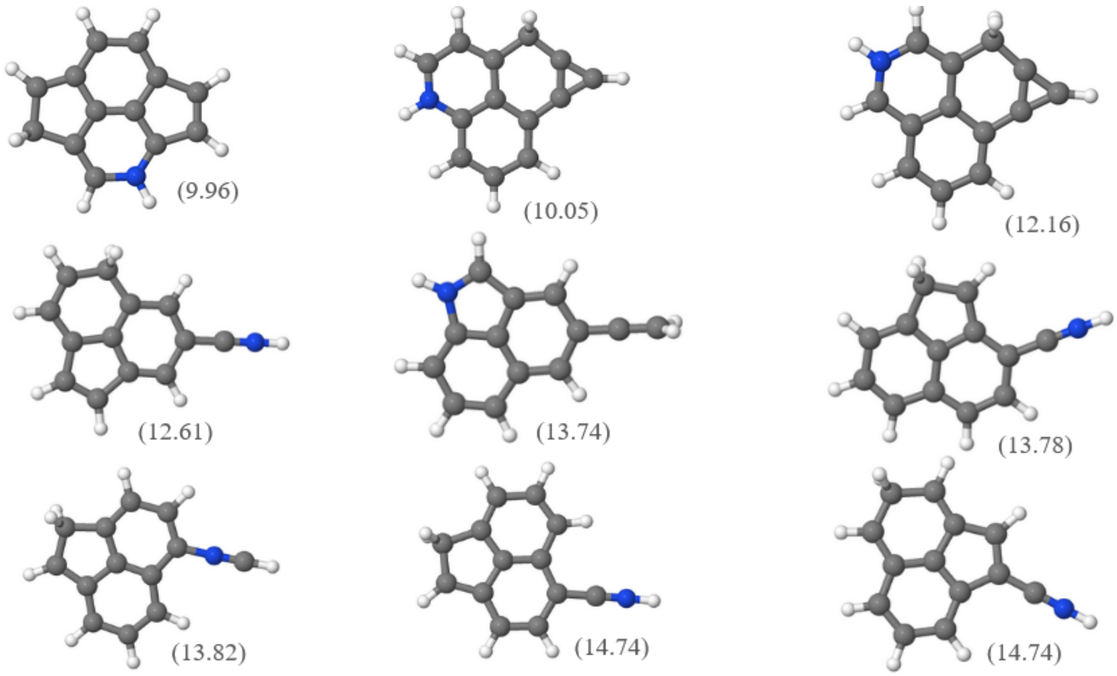

Figure S6: Other structures obtained for phenantridine dication applying the automated reaction path search. The numbers in parenthesis are the relative energies (ΔE) in kcal mol^{-1}.

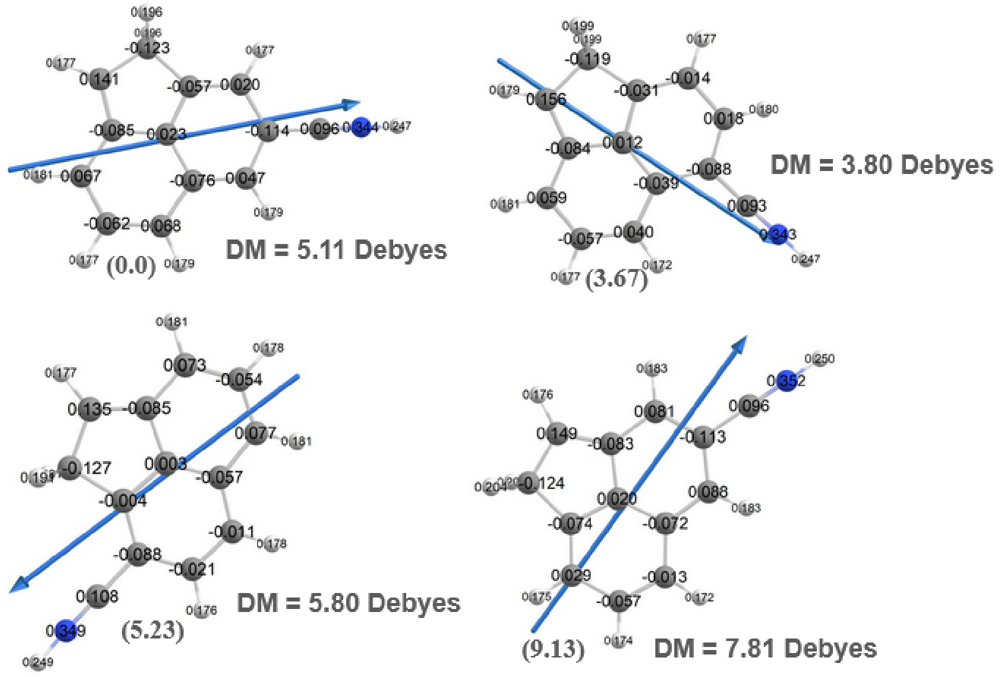

Figure S7: Dipole moment vector and charge distribution for the four most stable G.M candidate for phenantridine^{2+}.

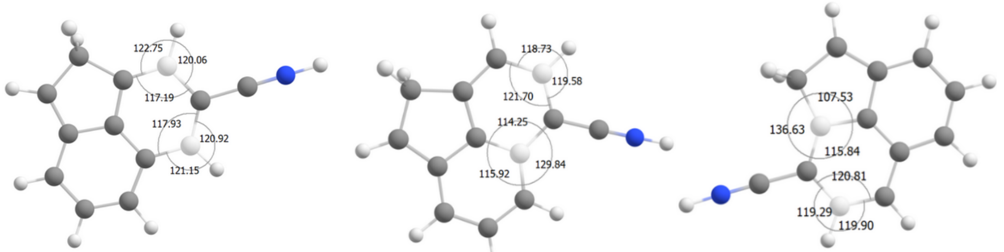

(0.0) (3.67) (5.23)

Figure S8: Bond angles for the carbons adjacent to the carbon of the C−N−H group in the three most stable phenantridine^{2+} isomers.

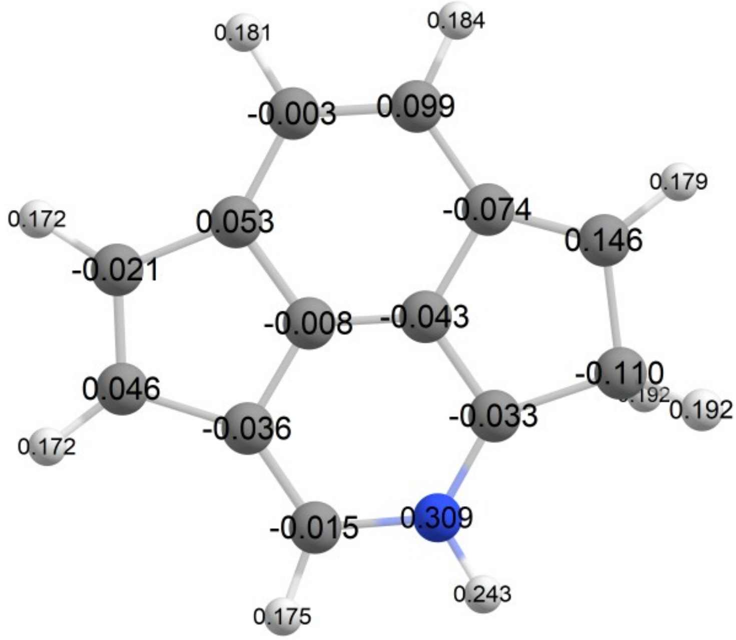

Figure S9: charge distribution for the less stable G.M candidate for phenantridine^{2+}.

3 Simulated infrared spectra

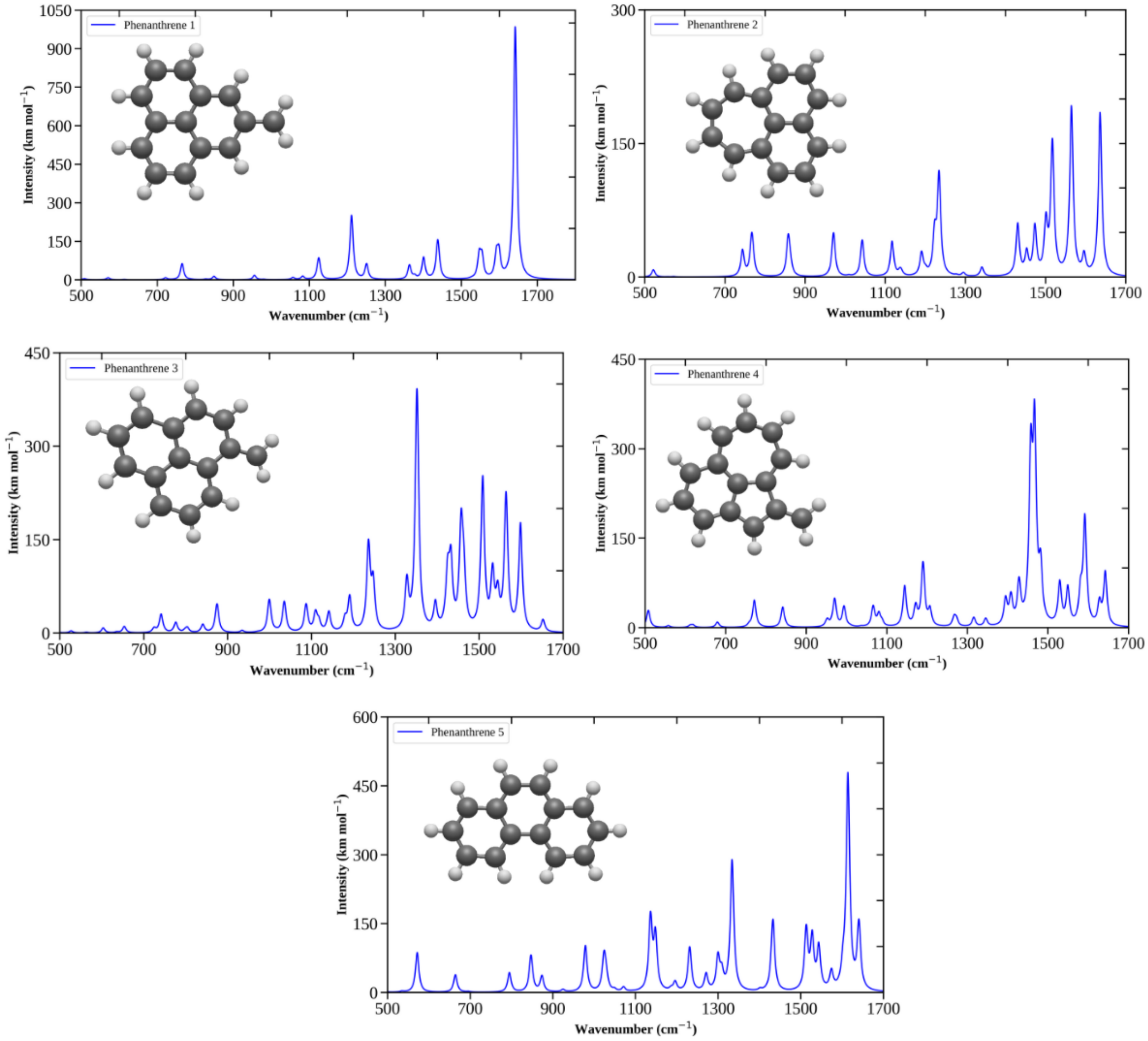

Figure S10: Simulated infrared spectra in the C–H stretching region.

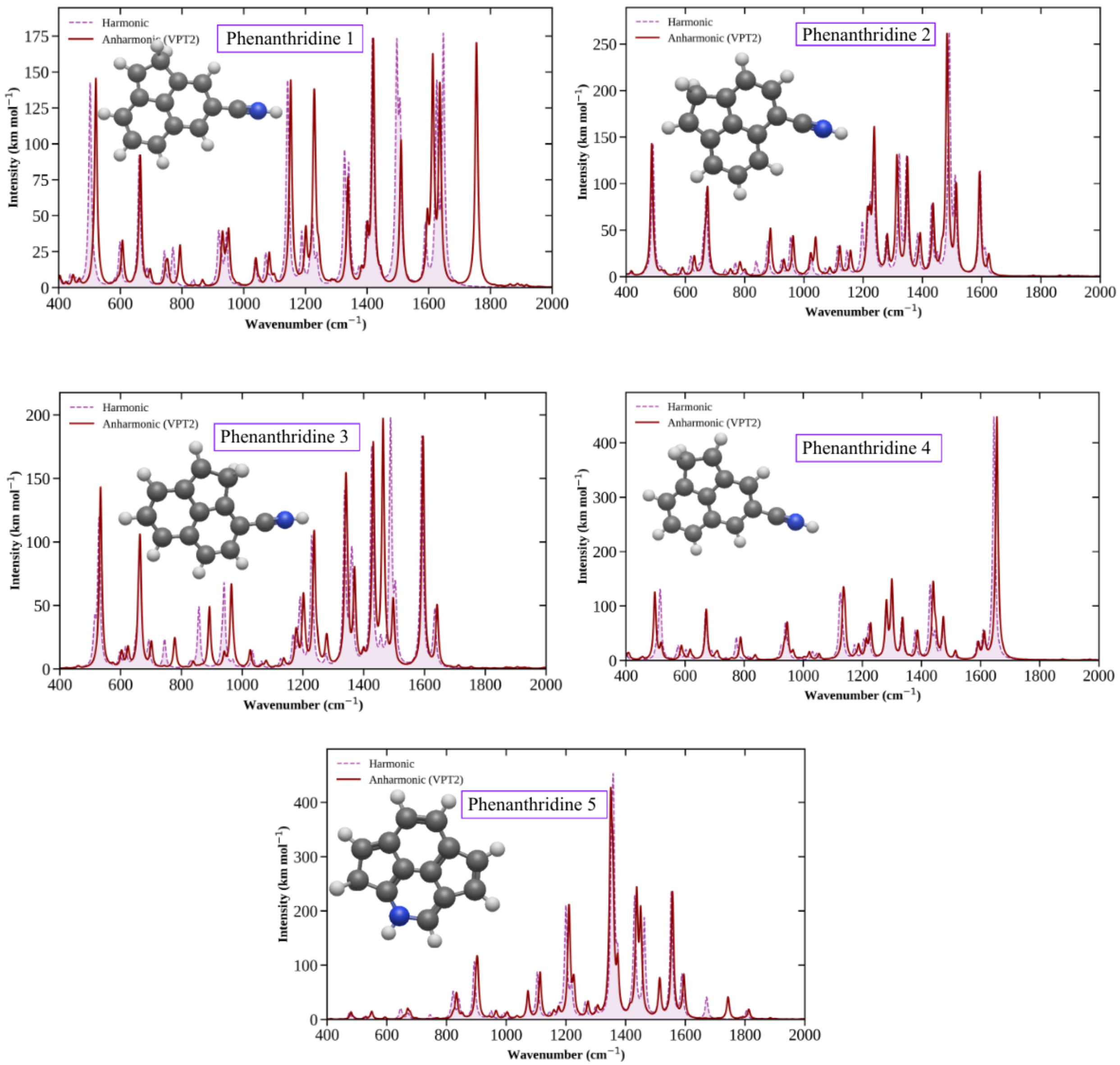

Figure S11: Simulated infrared spectra in the C–H stretching region. Red lines are the spectra obtained with VPT2 (Anharmonic). Purple dashed lines are the harmonic spectra with dual scalling factor.

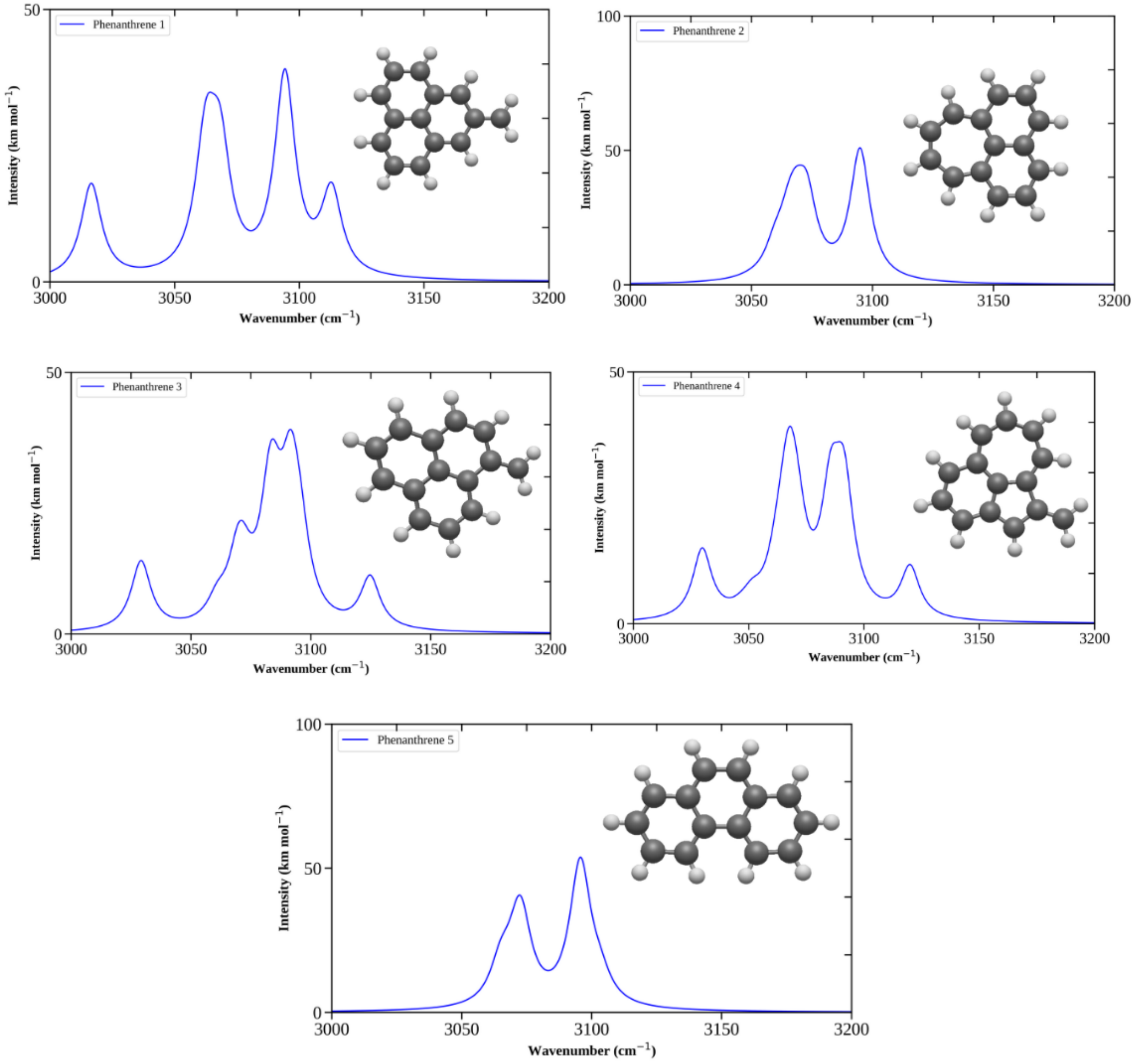

Figure S12: Simulated infrared spectra in the C–H stretching region.

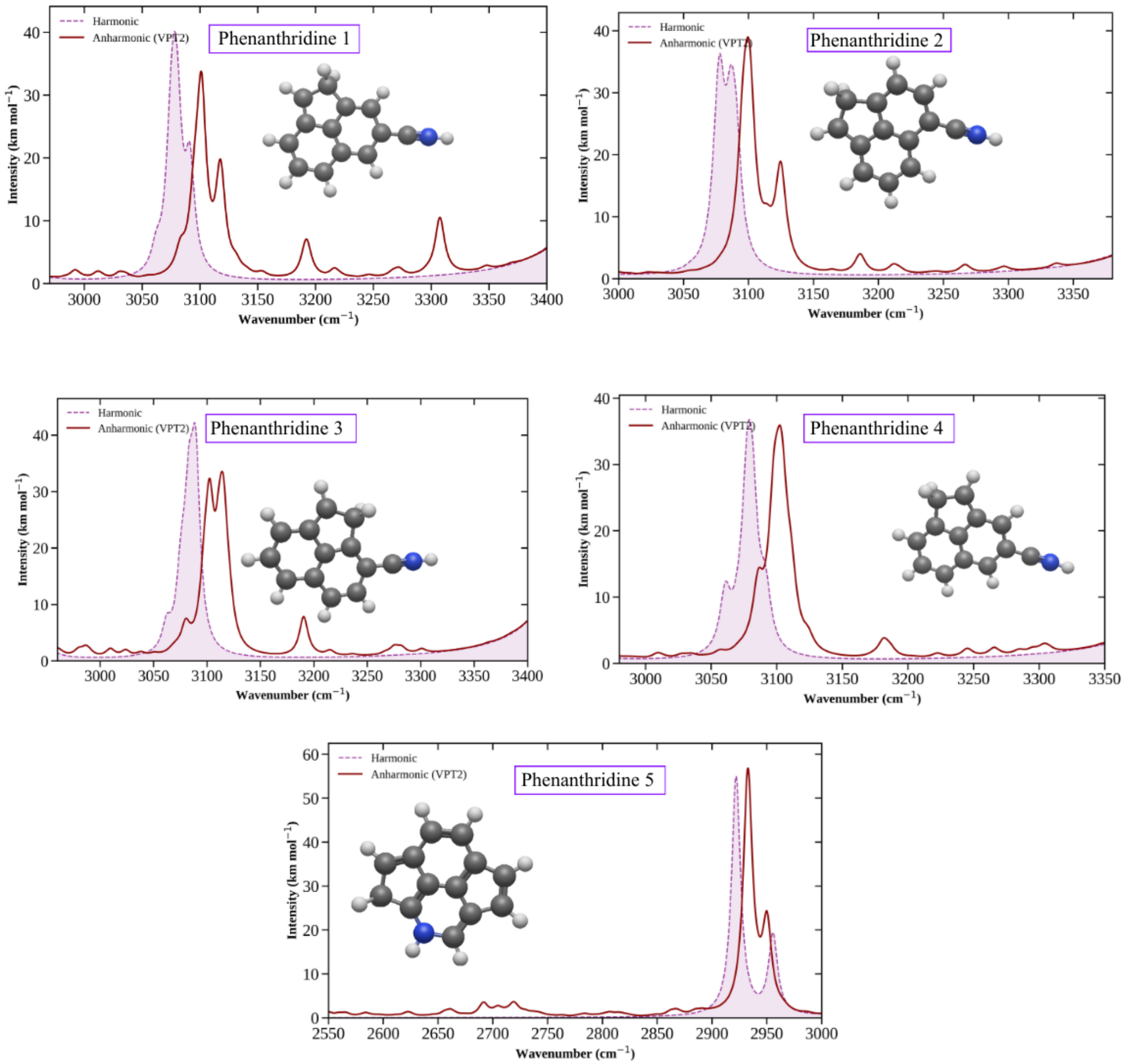

Figure S13: Simulated infrared spectra in the C–H stretching region. Red lines are the spectra obtained with VPT2 (Anharmonic). Purple dashed lines are the harmonic spectra with dual scaling factor.

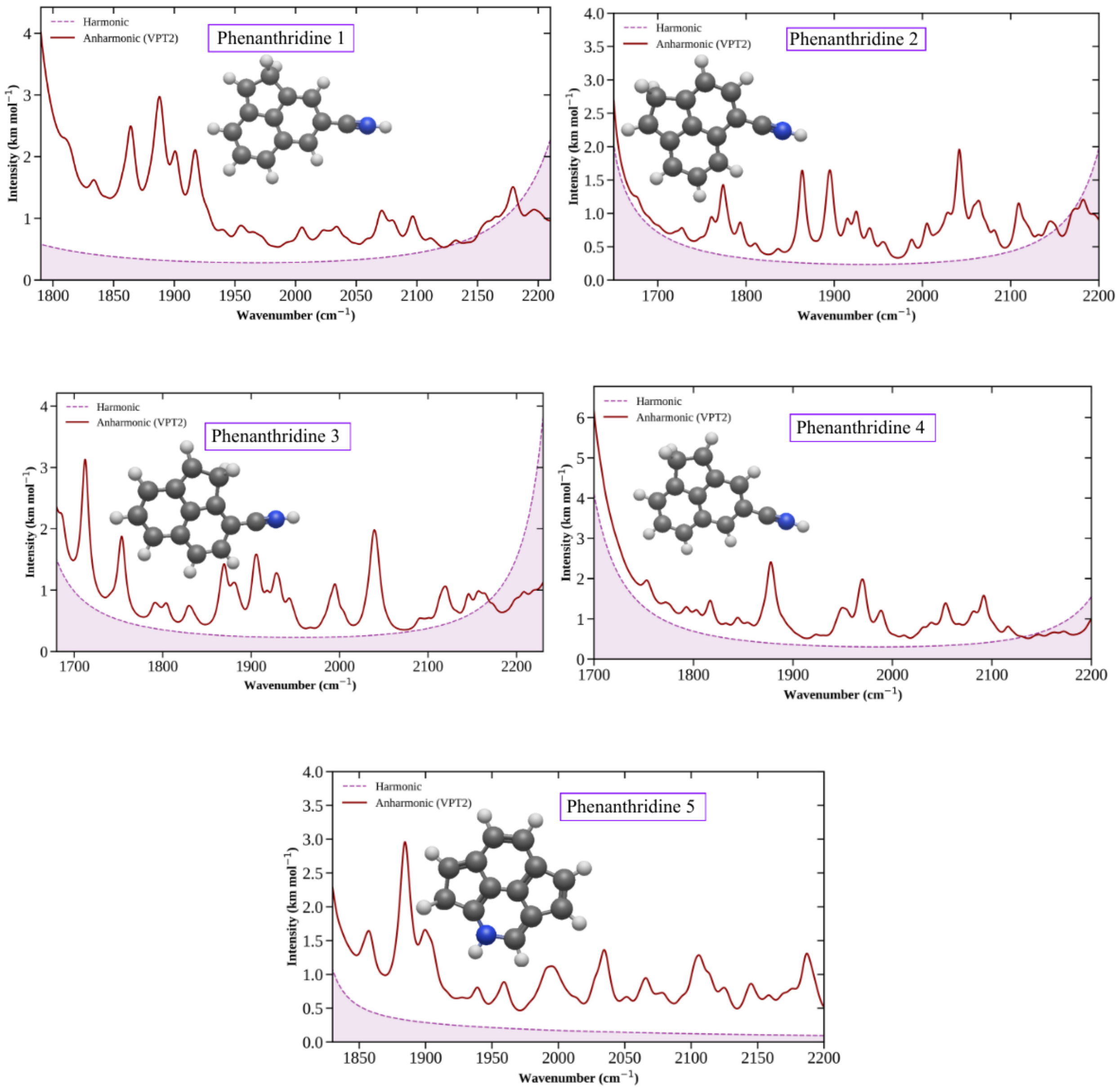

Figure S14: Simulated infrared spectra in the 1800 - 2200 cm^{-1} overtone and combination bands region.

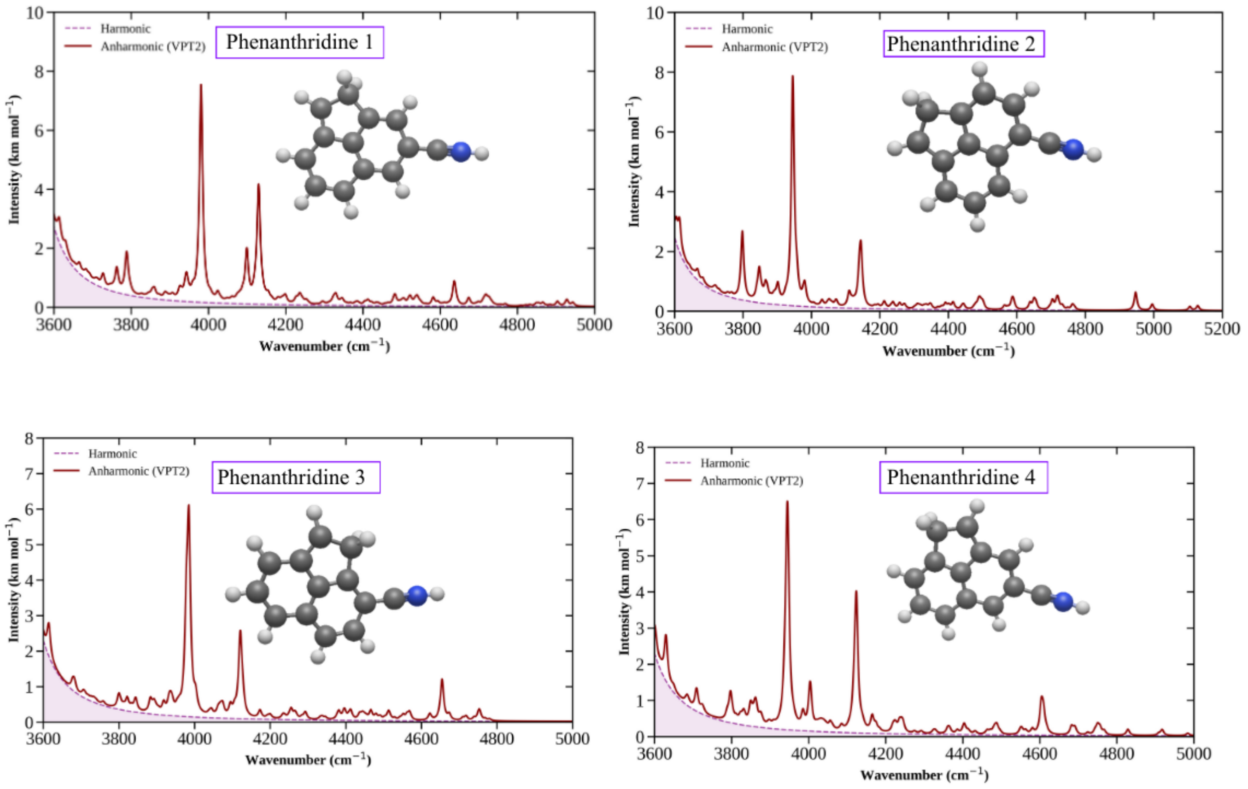

Figure S15: Simulated infrared spectra in the 3600 - 5000 cm^{-1} overtone and combination bands region.

4 Ionization Potentials

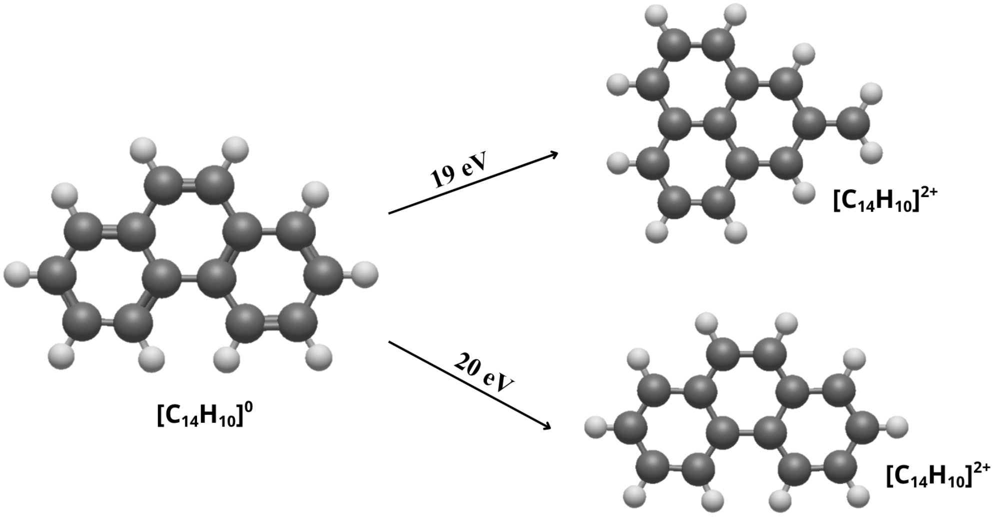

Figure S16: Adiabatic (19 eV) and Vertical (20 eV) Ionization Potentials for Phenanthrene.

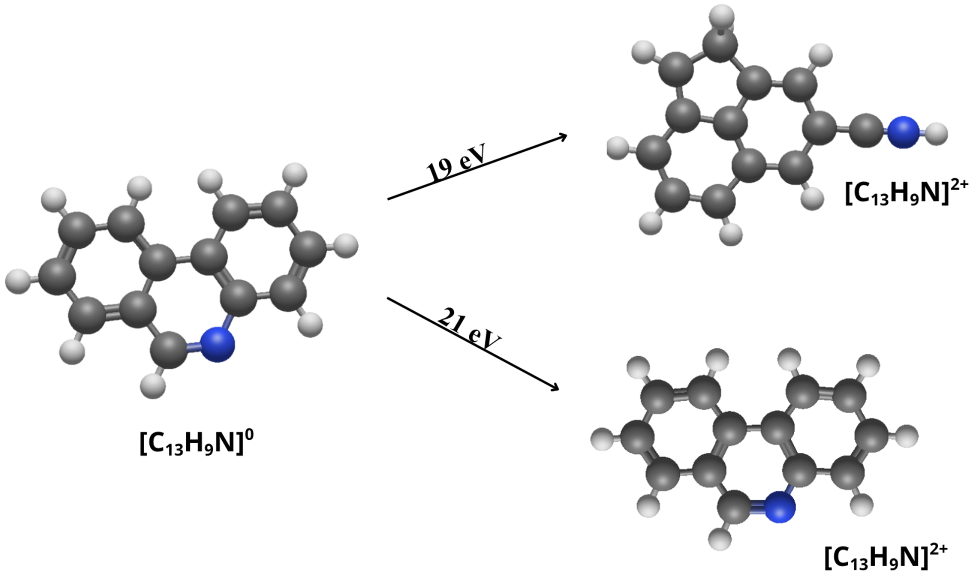

Figure S17: Adiabatic (19 eV) and Vertical (21 eV) Ionization Potentials for Phenanthridine.

5 Cartesian Coordinates of Phenanthrene dications

Isomer 1

C	-2.385906213605	0.027515831598	0.005204466553
C	-0.281768853878	1.266610878043	0.004876615386
C	0.412178911592	0.021463716126	0.004814911270
C	0.453174823370	-2.441933726495	0.004957441038
C	-3.752802086141	0.030472955992	0.005361919741
C	-1.659866644538	1.263150069152	0.005055027488
C	1.838387326270	2.471735515379	0.004517417085
C	0.463856367893	2.484671004115	0.004738899366
C	2.502289180532	-1.217442041728	0.004489571105
C	1.816337069589	0.018434514124	0.004565533762
C	2.507637519506	1.251335925361	0.004411151082
C	1.827755867349	-2.434930294841	0.004697313660
H	-2.210987284887	2.195942075519	0.005084070615
H	-4.317245883841	0.956118869205	0.005369170769
H	-0.072586543063	3.425172081320	0.004811124587
H	-4.321245669700	-0.892722968001	0.005481486348
H	-0.087330127017	-3.380105943151	0.005126319993
H	3.585814172595	-1.211234651813	0.004268804467
H	3.591124245437	1.240450617473	0.004211010264
H	2.382204344359	-3.361752316608	0.004651387329
C	-0.287169965937	-1.220666787929	0.004998752838
C	-1.665248514173	-1.211251243812	0.005191719036
H	2.396816412012	3.396163373955	0.004416857065
H	-2.220406837611	-2.141646460242	0.005329118603

Isomer 2

C	2.888542854235	-0.658918307003	-0.007266572628
C	0.407067312144	1.297065345752	-0.006798716795
C	-0.236380983648	0.021447407862	-0.006819470094
C	-0.398499015295	-2.449941011130	-0.006873232749
C	2.885677486501	0.714798792756	-0.007196256543
C	1.775072077077	1.568271513585	-0.006959769235
C	-1.771510635705	2.464265930050	-0.006495394335
C	-0.408797446013	2.492149920217	-0.006614791377
C	-2.390593141130	-1.184814111156	-0.006696261051
C	-1.661407041615	0.018486278202	-0.006700187413
C	-2.395608296136	1.218743201914	-0.006557944708
C	-1.761316051017	-2.427720366554	-0.006766258848
H	2.029200494528	2.621468407686	-0.006912307044
H	3.856954074060	-1.143988253764	-0.007446805525
H	0.103484033682	3.445231896640	-0.006579791523
H	3.852053385229	1.203910688133	-0.007325923385
H	0.117761408702	-3.400876033355	-0.006930118758
H	-3.472686831899	-1.132440910056	-0.006624127323
H	-3.477476011698	1.161869966568	-0.006484249016
H	-2.336853603927	-3.341726629267	-0.006737969372
C	0.412381371454	-1.251477183077	-0.006930159570
C	1.781503868033	-1.517006132840	-0.007116149300
H	-2.350846963349	3.375867962788	-0.006364101775
H	2.039969448572	-2.569143036586	-0.007174451724

Isomer 3

C	1.803753707987	1.771971737505	0.001909580597
C	0.948878139555	-0.557864117586	0.001846042127
C	-0.341880381150	-0.032249774008	0.001817096513
C	-1.919840158887	1.878552323438	0.001752045006
C	3.374957740319	-0.014619434947	0.002168978373
C	2.058635352651	0.374337933387	0.001942852307
C	0.031168065770	-2.821899799970	0.001726714520
C	1.110511809495	-1.976385173744	0.001772553702
C	-2.754547059666	-0.366387477431	0.001784303815
C	-1.462929473991	-0.904657561200	0.001793290531
C	-1.264006969075	-2.292592513355	0.001764352254
C	-2.981515556940	1.030123980646	0.001755117199
H	3.691295037513	-1.049364949783	0.002288424464
H	2.642258224426	2.456054426357	0.001972483627
H	2.099386129599	-2.411429136787	0.001734135057
H	4.165235029462	0.727222731307	0.002265072987
H	-2.072087314835	2.950155577369	0.001719229646
H	-3.604630760064	-1.038754549374	0.001791482822
H	-2.119873718695	-2.955871005674	0.001756098937
H	-3.993673454474	1.408209520850	0.001730377675
C	-0.560912872436	1.378581220099	0.001799240150
C	0.509383835064	2.265958258916	0.001824629349
H	0.178264212966	-3.892054150787	0.001666136056
H	0.334593532293	3.333090929536	0.001798109198

Isomer 4

C	1.420949633551	2.404171254810	-0.004840060257
C	-1.306570294896	0.824925619130	-0.004167035019
C	-0.307385493420	-0.157806142168	-0.004320156419
C	2.901183001532	-1.888610676356	-0.004462595350
C	0.124008543121	2.900800499036	-0.004537285956
C	-1.081302717895	2.211278527042	-0.004211746157
C	-3.009915541348	-0.970767376378	-0.003953314362
C	-2.668880070707	0.359904865933	-0.003954004264
C	0.490114738272	-2.316255747524	-0.004361744712
C	-0.674210285819	-1.544354704690	-0.004260504685
C	-2.009717619956	-1.949583259275	-0.004108254927
C	1.625804360104	-1.452804388143	-0.004486650604
H	-1.971021705584	2.830674968531	-0.004009009625
H	2.200863781538	3.156521528699	-0.005120030229
H	-3.455823043653	1.103735309925	-0.003808609683
H	0.040919628627	3.980284918094	-0.004577215369
H	3.750177505466	-1.217071192229	-0.004493733273
H	0.537189696114	-3.397526360483	-0.004329350183
H	-2.269240689973	-3.000834452675	-0.004105544347
H	3.121716208099	-2.948795267507	-0.004388692051
C	1.107080731927	-0.056953653201	-0.004523391243
C	1.877213462256	1.078517420214	-0.004796012557
H	-4.050608331600	-1.261112088734	-0.003824329085
H	2.952975924274	0.956189684511	-0.005016205111

Isomer 5

C	-1.430681234027	0.843218930954	0.000042183663
C	-0.738973994092	-0.426349839159	-0.000224178458
C	0.738974714694	-0.426350659580	-0.000207185729
C	1.430683815234	0.843217280920	-0.000313967925
C	-2.872182548420	0.888881108557	0.000334215202
C	-3.603485390990	-0.268699174410	0.000369694226
C	-2.915328498066	-1.478963015428	-0.000034387843
C	-1.495840332649	-1.560039721363	-0.000392579554
C	1.495839335720	-1.560041724891	0.000031131578
C	2.915328131126	-1.478966863952	0.000144638468
C	3.603487409517	-0.268704242013	0.000076221702
C	2.872185387424	0.888877147728	-0.000190609398
H	-3.358138209673	1.856368397341	0.000534163306
H	-4.683292144322	-0.250351200780	0.000665097297
H	-3.476760123600	-2.405405179249	-0.000016545782
H	-1.051341430019	-2.543588982820	-0.000700052440
H	1.051339870481	-2.543590705776	0.000152266752
H	3.476758640137	-2.405409898513	0.000275938296
H	4.683294069519	-0.250357350431	0.000218253400
H	3.358141610011	1.856364040334	-0.000256595818
C	0.727900216561	2.007633936235	-0.000372286846
C	-0.727896092002	2.007634878149	-0.000000347665
H	1.241268092183	2.961260513093	-0.000609792980
H	-1.241261964271	2.961262351871	0.000274782322

Isomer 6

C	-1.400332475295	1.185833746186	-0.008614804271
C	-0.670272323898	-0.022532025400	-0.008466227153
C	0.673499769224	-0.037723214395	-0.008461841429
C	1.435629828483	1.130559670431	-0.008581967518
C	-2.733569999075	0.801511813695	-0.008585509786
C	-2.870087282192	-0.702453727917	-0.008408968145
C	-0.760968976974	-2.413488956862	-0.008225499485
C	-1.432402252132	-1.190814958087	-0.008347480311
C	2.736798134093	-0.861766071757	-0.008396150586
C	1.403560877910	-1.246088476922	-0.008350315339
C	0.650786293665	-2.452283527937	-0.008232414927
C	2.873315156243	0.642199901673	-0.008494398232
H	-3.595758801863	1.458657527790	-0.008673551912
H	-3.447819889201	-1.036872049765	-0.878066744614
H	-1.301652711465	-3.349799932366	-0.008131224317
H	-3.447818077130	-1.036661160736	0.861331844023
H	3.451118057255	0.976479956063	-0.878155978994
H	3.598987490089	-1.518911138055	-0.008357022513
H	1.145598600156	-3.414930182679	-0.008145176870
H	3.450975279666	0.976545735152	0.861242864622
C	0.764196894508	2.353233777739	-0.008734670500
C	-0.647558322460	2.392028378990	-0.008756395725
H	1.304880022568	3.289544999498	-0.008840265331
H	-1.142371747791	3.354674516890	-0.008882831881

Isomer 7

C	-2.228371801146	0.519832902831	0.003002625808
C	0.012765446465	1.484865398654	0.002714182169
C	0.461324790749	0.158090991325	0.002731458483
C	0.590606606377	-2.144125273571	0.002833714938
C	-3.576967651094	0.734141635728	0.003123317818
C	-1.364602753022	1.661453225551	0.002852828042
C	2.361548669412	2.177242940343	0.002415420130
C	1.016231714149	2.501394144856	0.002551145772
C	1.911830821167	-1.583919965906	0.002650375558
C	1.833240719216	-0.182637197559	0.002591732464
C	2.791610857942	0.846884531080	0.002431147981
C	0.348128688885	-3.469713579376	0.002939084150
H	-1.804471928384	2.651359221408	0.002848798769
H	-3.989588390337	1.735964052056	0.003109918397
H	0.719308381996	3.542884562593	0.002531103260
H	-4.283174045697	-0.088130455907	0.003235631943
H	1.163338100113	-4.182200749800	0.002882593467
H	2.819357458490	-2.173554214048	0.002574393538
H	3.850839959333	0.622230346932	0.002320388787
H	-0.656195199931	-3.873493043946	0.003084977277
C	-0.354144269736	-0.997601558613	0.002882876300
C	-1.698234181848	-0.834705933104	0.003019558149
H	3.096236562785	2.969196494473	0.002292348320
H	-2.396264425819	-1.661478088529	0.003137905372

Isomer 8

C	-1.080825830047	-2.642454483848	-0.004310038570
C	2.273993130052	-1.124251130170	-0.004039497016
C	0.020494652129	-0.077883797009	-0.004197816387
C	-3.286249014826	1.484861160046	-0.004350382234
C	0.274865958326	-2.502068611520	-0.004186607608
C	0.886797528086	-1.204983842077	-0.004136118505
C	2.816325578987	1.350355914326	-0.004112445144
C	3.119969430842	0.005503919062	-0.004040877996
C	-0.923614032136	2.023966511248	-0.004243246764
C	0.327790195752	1.319989434509	-0.004185893475
C	1.550582392753	1.963253045550	-0.004160897031
C	-1.981520337768	1.097591867695	-0.004305801625
H	2.795442551017	-2.075104119352	-0.003972716958
H	-1.524209240469	-3.627739888987	-0.004367060645
H	4.177740699100	-0.228454943404	-0.003986301430
H	0.912917316173	-3.375088147228	-0.004138236869
H	-4.103348384487	0.773202321160	-0.004391241645
H	-1.031338398177	3.100868619846	-0.004237606332
H	1.540310123369	3.046985723919	-0.004186164523
H	-3.550004221178	2.535702761521	-0.004342466438
C	-1.373011898387	-0.249250531354	-0.004288910674
C	-1.926501988783	-1.504540464003	-0.004360453323
H	3.663447203023	2.025052462158	-0.004118912341
H	-2.999213173441	-1.648550477967	-0.004455570255

Isomer 9

C	1.885751089518	-1.722550138197	-0.002368409110
C	0.407264832624	0.258064115495	-0.002389869122
C	-1.062877632199	0.451954158156	-0.002344317918
C	-1.664003248341	-0.867966155204	-0.002189833546
C	2.971597481891	-0.855742000512	-0.002442794762
C	3.889134727769	1.363757821580	-0.002470409201
C	2.783422776587	0.556817267232	-0.002464801794
C	1.452845567152	1.116716659719	-0.002449799839
C	-1.849314979693	1.556191133470	-0.002445901695
C	-3.253249563530	1.356645569282	-0.002376246904
C	-3.864006321030	0.092132925073	-0.002195505730
C	-3.085960230935	-1.033852713184	-0.002099538303
H	3.980313883705	-1.248439570319	-0.002470222921
H	4.889520585141	0.946822674485	-0.002474516013
H	3.804902000759	2.444537061391	-0.002462198328
H	1.329287255652	2.191285222612	-0.002476195490
H	-1.453756031512	2.561360030151	-0.002584396352
H	-3.890894758703	2.232590890554	-0.002464423350
H	-4.941708782514	0.022092284203	-0.002139068664
H	-3.519719486568	-2.024992390413	-0.001960969583
C	-0.689076715168	-1.816997324284	-0.002193744978
C	0.632333736810	-1.148699621882	-0.002324757988
H	-0.831359822471	-2.889654731162	-0.002114727801
H	2.026348733132	-2.793968352783	-0.002336425097

Isomer 10

C	-1.149389512470	1.164301033121	0.000077151140
C	0.000331326426	-1.115682996491	0.000196515945
C	0.000340807886	0.281109868931	0.000171185187
C	1.150083620996	1.164285578271	0.000236732328
C	-2.496517525065	0.890445294581	-0.000108047760
C	-3.117191323194	-0.360174578993	-0.000006976756
C	-2.521660689472	-1.620947305243	0.000343391598
C	-1.183863238543	-1.931180263632	0.000397381235
C	1.184514070172	-1.931197080668	0.000012938067
C	2.522316155624	-1.620983343329	-0.000098428414
C	3.117863537941	-0.360218624854	0.000102264862
C	2.497207430116	0.890410311092	0.000279961472
H	-3.156874287632	1.749026847843	-0.000302533524
H	-4.201394222719	-0.348814091072	-0.000166527571
H	-3.198571124260	-2.465722322994	0.000517866502
H	-0.984618406961	-2.995241933113	0.000599600237
H	0.985253954697	-2.995255893663	-0.000070599977
H	3.199215134713	-2.465767783649	-0.000291074560
H	4.202066571934	-0.348873046942	0.000100873962
H	3.157576447953	1.748982511345	0.000399375238
C	0.666686375811	2.551379112638	0.000245298592
C	-0.665973511025	2.551387913336	0.000112107553
H	1.322293167004	3.408332241316	0.000319333930
H	-1.321570227052	3.408348884508	0.000046577655

Isomer 11

C	1.395432994994	-0.846246399620	-0.002101029318
C	0.916676049174	0.504425687399	-0.002010892557
C	-0.521597444571	0.691760990379	-0.002032098703
C	-1.412819613588	-0.473356021164	-0.002093392390
C	2.786977542788	-1.099590471496	-0.002080723302
C	3.693896781598	-0.044835991907	-0.001962366834
C	3.215936800592	1.248521139367	-0.001871511498
C	1.821405236338	1.530586378634	-0.001899207380
C	-1.283304986460	1.841340633993	-0.002013013406
C	-2.644244204271	1.474192607410	-0.002034893815
C	-2.794711028563	0.057267428379	-0.002071641478
C	-3.973103282542	-0.590581066622	-0.002059185291
H	3.138975109836	-2.123378747439	-0.002156185707
H	4.756789183965	-0.237579894255	-0.001941389009
H	3.910885534956	2.077946008424	-0.001777767980
H	1.501822303197	2.563120263516	-0.001821627784
H	-0.927187071185	2.859225870873	-0.001986369179
H	-3.470835832839	2.173466279336	-0.002021814205
H	-4.905564793284	-0.040588097825	-0.002030587440
H	-4.041038174419	-1.670874235722	-0.002070686836
C	-0.928356476525	-1.726372281146	-0.002181644959
C	0.504195455023	-1.904482405400	-0.002186360339
H	-1.564950304636	-2.599901546351	-0.002248417979
H	0.892518029071	-2.916626422532	-0.002252894939

Isomer 12

C	-4.550646899017	-0.381798211927	-0.003922629975
C	0.250125037751	-1.189167323104	-0.003560822839
C	0.767812441480	0.109149699414	-0.003694845083
C	0.021249683002	1.297350203504	-0.003888921509
C	-1.922517069612	-0.119304090735	-0.003818780702
C	1.199873075627	-2.250502855650	-0.003373793093
C	2.568957466163	-2.005909111168	-0.003327638269
C	-1.142670217161	-1.300115077014	-0.003623637252
C	2.326360337850	1.747246561119	-0.003818596906
C	2.165777171255	0.375640924766	-0.003652225129
C	3.074063109951	-0.710185620384	-0.003465222106
C	1.009694474266	2.436733252239	-0.003984385340
H	-5.105823872813	-0.436686352055	-0.939854947647
H	0.849542138008	-3.275773412412	-0.003263805113
H	3.252043226395	-2.842782423882	-0.003182918202
H	-1.636810064953	-2.262942680023	-0.003526832607
H	3.271628847901	2.275977163222	-0.003833400295
H	0.927566241692	3.098767418872	-0.874320276153
H	4.143487506076	-0.541145114577	-0.003430085607
H	0.927465961815	3.099011521076	0.866155539601
C	-1.334850813353	1.202755334694	-0.003957410621
C	-3.284292512951	-0.256131346439	-0.003874347139
H	-1.989331000259	2.063794047922	-0.004108912142
H	-5.105920768542	-0.436431877920	0.931967383013

Isomer 13

C	2.006556336028	-1.637250163647	-0.002428271470
C	1.218961928583	0.694455076887	-0.002286081636
C	-0.128507529857	0.189463914471	-0.002278646503
C	-1.691700054868	-1.742752992272	-0.002357941592
C	3.592217040546	0.244031668908	-0.002449712587
C	2.297902424681	-0.215087828489	-0.002377157451
C	-0.108681240471	2.526681307316	-0.002139495547
C	1.177414030073	2.151474038146	-0.002195396582
C	-3.213420619017	0.257506422329	-0.002222070623
C	-2.359838470631	1.364785645110	-0.002168890662
C	-0.988741344747	1.318320105385	-0.002194945051
C	-2.913835179079	-1.114581246322	-0.002306537033
H	3.828489304631	1.300555163650	-0.002428150353
H	2.835905181381	-2.331368462883	-0.002483648198
H	2.029758170560	2.810790309578	-0.002169660393
H	4.430078653095	-0.444788991146	-0.002542204339
H	-1.723531854884	-2.826197315259	-0.002416165788
H	-4.272348908157	0.491317200404	-0.002192323372
H	-2.830580506314	2.339969457583	-0.002102057818
H	-3.775371995869	-1.770431327549	-0.002331533610
C	-0.380941476662	-1.177659103874	-0.002348441848
C	0.736122950026	-2.089065348630	-0.002413591020
H	-0.487170806201	3.537927464266	-0.002063200216
H	0.536654089350	-3.151108218941	-0.002455752810

Isomer 14

C	1.928371514688	0.598730074271	0.001944255123
C	0.693720457674	-0.111812009665	0.002104826665
C	-0.537061200627	0.502513591345	0.002474225461
C	-0.523452227898	1.918860152821	0.003110284701
C	2.945496484201	-0.335902350325	0.002181152830
C	2.399455706591	-1.723051709486	0.002671231824
C	-0.165964987485	-2.363563360286	0.002774672455
C	0.907269276899	-1.507366118369	0.002431388042
C	-2.961485286293	0.042994491140	0.002311954533
C	-1.671789098157	-0.398272188393	0.002545659967
C	-1.450172237955	-1.796123655742	0.002661705822
C	-4.173655434639	0.435547090068	0.002051996749
H	4.007042474375	-0.118657374567	0.002247396025
H	2.759046752390	-2.284937508845	0.872989591858
H	-0.052251838692	-3.438406079738	0.003113030831
H	2.759477398374	-2.286069288056	-0.866709523297
H	-4.706406071444	0.603547555049	0.937431731295
H	-1.457670442298	2.466535440469	0.003839417168
H	-2.315461932136	-2.445758731621	0.002704092526
H	-4.706143350538	0.603317846917	-0.933517692427
C	0.668562847000	2.638274160081	0.002973615408
C	1.910460746786	2.006912897223	0.002225002879
H	0.623588909675	3.717885202465	0.003532955001
H	2.824778741161	2.585445052142	0.002058125318

Isomer 15

C	-2.244553911615	-1.082636354537	0.006726870622
C	-1.248112584474	1.303081239656	0.006699130604
C	0.728875648265	-0.185082263042	0.006093904340
C	1.274536014447	-2.470005814781	0.005794432723
C	-3.583823982803	0.900042007497	0.007199082952
C	-2.319950182545	0.358811381394	0.006865188322
C	0.989352276350	2.230876160281	0.006232739976
C	0.123909618301	1.082972960530	0.006348513810
C	2.427551884688	-1.760233662916	0.005559317125
C	2.108919879268	-0.311384707188	0.005745222629
C	2.349109190746	2.088877189164	0.005880721519
C	2.927938072297	0.801361734054	0.005629894053
H	-3.744133187289	1.971894639280	0.007336080824
H	-3.213906251592	-1.568602237701	0.006905864966
H	-1.558478972158	2.342160526844	0.006869950017
H	-4.470254784936	0.275866029857	0.007336283245
H	1.175312366561	-3.543373687490	0.005743131007
H	3.428517351835	-2.167447637639	0.005280216891
H	2.987296120289	2.960594157413	0.005796513381
H	4.005591857628	0.697691737167	0.005354002966
C	0.173129660015	-1.554290835495	0.006127742618
C	-1.166891902008	-1.930822780982	0.006414867341
H	0.545778106925	3.217314829160	0.006430939947
H	-1.379563687486	-2.992098626484	0.006382137490

6 Cartesian Coordinates of Phenanthrene dications

Isomer 1

C	-1.033716	-2.101199	0.069215
C	-0.080266	-1.046696	0.065470
C	-0.596740	0.244404	0.063249
C	2.073892	0.003851	0.059972
C	-2.403247	-1.852149	0.070592
C	-2.145714	1.891660	0.061627
C	-2.909103	-0.556750	0.068340
C	-1.995632	0.521258	0.064586
C	0.156282	1.422927	0.059453
C	1.516773	1.321320	0.057697
C	-0.821299	2.572422	0.058157
C	1.323203	-1.164329	0.063757
H	5.632440	-0.297992	0.055590
H	-3.086266	-2.689066	0.073468
H	-3.977754	-0.385202	0.069443
H	-0.731228	3.234071	0.927330
H	2.167852	2.184776	0.054734
H	-3.086168	2.428478	0.061709
H	-0.733696	3.229938	-0.814402
H	1.803458	-2.133546	0.065329
C	3.489795	-0.120919	0.058239
N	4.624664	-0.213977	0.056839
H	-0.691136	-3.128952	0.071058

Isomer 2

C	-0.576550	-1.855532	-0.031223
C	-0.591961	-0.439393	-0.030063
C	0.645653	0.178702	-0.030165
C	-1.702400	0.466770	-0.028776
C	0.613157	-2.577234	-0.032393
C	2.898103	0.390888	-0.031086
C	1.855040	-1.947520	-0.032499
C	1.876514	-0.540087	-0.031358
C	0.862977	1.565421	-0.029125
C	-0.213984	2.408300	-0.027885
C	2.356611	1.776314	-0.029682
C	-1.509662	1.839458	-0.027720
H	-5.084723	-0.666463	-0.028135
H	0.565347	-3.656509	-0.033242
H	2.768188	-2.527823	-0.033424
H	2.724210	2.334295	-0.899198
H	-0.111878	3.484333	-0.027043
H	3.958280	0.168678	-0.031781
H	2.725066	2.332868	0.840385
H	-2.366903	2.498858	-0.026755
C	-3.038819	-0.002990	-0.028533
N	-4.118985	-0.365889	-0.028327
H	-1.506167	-2.412119	-0.031222

Isomer 3

C	1.685935	-0.467804	0.043408
C	0.683671	0.488520	0.045059
C	-0.617873	0.001813	0.043586
C	0.605167	1.997658	0.048206
C	1.379047	-1.857738	0.040337
C	-2.373618	-1.603450	0.039608
C	0.074904	-2.290468	0.038968
C	-0.969545	-1.340010	0.040616
C	-2.946193	0.760109	0.044420
C	-1.569515	1.070614	0.045543
C	-3.317507	-0.583647	0.041444
C	-0.870344	2.252237	0.048266
H	5.151772	0.403709	0.046427
H	2.185321	-2.577196	0.039099
H	-0.139168	-3.350075	0.036674
H	1.068910	2.468308	-0.825445
H	-3.697965	1.538713	0.045826
H	-2.716574	-2.631190	0.037356
H	-4.366450	-0.842828	0.040566
H	-1.293582	3.248649	0.050227
C	3.060606	-0.096759	0.044674
N	4.165378	0.177336	0.045628
H	1.068364	2.464648	0.924101

Isomer 4

C	0.989188	2.131008	0.072831
C	0.056136	1.048172	0.067763
C	0.593210	-0.253289	0.065016
C	-2.120209	-0.057681	0.060047
C	2.327596	1.851689	0.074764
C	2.083191	-2.039575	0.062794
C	2.828160	0.518487	0.071851
C	1.947482	-0.539543	0.066925
C	-0.192464	-1.443618	0.059891
C	-1.592811	-1.347889	0.057270
C	0.683909	-2.520656	0.058529
C	-1.325267	1.128045	0.065200
H	-5.673733	0.321351	0.053863
H	3.039336	2.664490	0.078619
H	3.899242	0.362981	0.073579
H	2.617804	-2.452043	0.929475
H	-2.231783	-2.221038	0.053350
H	2.621323	-2.446947	-0.804121
H	0.410136	-3.568245	0.054906
H	-1.819074	2.092470	0.067066
C	-3.535535	0.090850	0.057580
N	-4.667070	0.212961	0.055619
H	0.638467	3.153599	0.075105

Isomer 5

C	-1.305638	-0.892095	0.029272
C	-0.487854	0.218318	0.026042
C	0.838276	0.106469	0.024522
C	1.504360	-1.112163	0.026051
C	-2.731213	-0.363797	0.030437
C	-0.320743	2.615714	0.022143
C	-2.505629	1.147305	0.027619
C	-1.166858	1.462135	0.025027
C	1.703835	1.239830	0.021759
C	3.044822	0.655167	0.021362
C	1.087998	2.495296	0.020553
C	2.949157	-0.702761	0.023828
H	-1.234033	-2.928746	0.033097
H	-3.309356	-0.669311	0.907868
H	-3.311828	-0.672203	-0.844351
H	-0.750464	3.609858	0.021227
H	-3.339537	1.838728	0.027856
H	3.969157	1.210607	0.019479
H	1.676137	3.402773	0.018511
H	3.786039	-1.384573	0.024194
C	0.703759	-2.221315	0.029212
N	-0.690205	-2.070784	0.030702
H	1.042724	-3.246615	0.030826

Isomer 6

C	1.461729	-0.988728	-0.038052
C	0.722820	0.175448	-0.035758
C	-0.612523	0.169331	-0.036761
C	-1.365260	-1.002885	-0.040220
C	2.893903	-0.545862	-0.035846
C	0.840754	2.553722	-0.029723
C	2.914166	0.805548	-0.032479
C	1.513345	1.338020	-0.032248
C	-1.348752	1.377194	-0.034321
C	-2.678020	0.985035	-0.036309
C	-0.580238	2.578437	-0.030757
C	-2.811628	-0.520803	-0.040157
H	1.240458	-3.014272	-0.043169
H	3.754595	-1.196058	-0.036779
H	3.806289	1.412233	-0.030270
H	1.369737	3.496517	-0.026960
H	-3.391050	-0.841864	-0.912710
H	-3.545106	1.635456	-0.035369
H	-1.073420	3.543062	-0.028747
H	-3.392452	-0.846230	0.829839
C	-0.642066	-2.162449	-0.042520
N	0.761383	-2.118493	-0.041337
H	-1.053986	-3.160646	-0.045291

Isomer 7

C	-1.294236	0.493392	0.031774
C	0.048856	0.090737	0.029348
C	1.031127	1.145595	0.026291
C	0.352925	-1.308545	0.030071
C	-1.654366	1.860457	0.031134
C	3.672513	0.242290	0.021785
C	-0.688800	2.825688	0.028157
C	0.675575	2.468949	0.025707
C	2.700283	-0.695377	0.024578
C	1.785234	-1.842588	0.027491
C	2.360213	0.627247	0.024055
C	-0.683442	-2.212407	0.033211
H	-3.236567	-0.174124	0.036551
H	-2.701268	2.136349	0.033019
H	-0.968526	3.869206	0.027667
H	1.436826	3.236613	0.023365
H	1.970748	-2.475843	0.899033
H	4.720804	0.506238	0.019485
H	1.967058	-2.477284	-0.843780
H	-0.500428	-3.277233	0.033915
C	-2.000619	-1.767168	0.035576
N	-2.266379	-0.471392	0.034815
H	-2.844433	-2.442753	0.038066

Isomer 8

C	0.796795	-2.071086	0.062863
C	-0.004988	0.188943	0.052836
C	-1.024242	1.197779	0.046352
C	1.334324	0.641532	0.055629
C	-1.679177	-1.821258	0.053942
C	-3.631264	0.202421	0.041223
C	-0.266028	-1.223724	0.056597
C	-2.627670	-0.700409	0.047312
C	-0.713850	2.540352	0.042912
C	0.627052	2.963087	0.045792
C	-2.332603	0.633649	0.043995
C	1.627815	2.034424	0.052014
H	2.834982	-2.261391	0.070056
H	-1.849165	-2.456951	-0.817946
H	0.701449	-3.146900	0.066114
H	-1.854781	-2.451337	0.928792
H	0.857474	4.018180	0.043072
H	-4.687728	0.430339	0.037146
H	-1.510224	3.272622	0.037970
H	2.662956	2.349799	0.054295
C	2.365609	-0.303313	0.062057
N	2.073874	-1.590096	0.065392
H	3.411508	-0.028750	0.064491

Isomer 9

C	-1.068536	-1.930303	0.044808
C	-0.071223	-0.824550	0.038388
C	-0.555548	0.447244	0.032832
C	2.100290	0.204381	0.031617
C	-2.480544	-1.500476	0.044026
C	-2.038263	2.245601	0.025901
C	-2.927656	-0.198602	0.038113
C	-1.966728	0.806512	0.032497
C	0.205636	1.623425	0.026633
C	1.563893	1.529815	0.025773
C	-0.775991	2.740808	0.022426
C	1.334337	-0.952901	0.037777
H	5.656631	-0.150205	0.030649
H	-3.213786	-2.299464	0.048641
H	-3.985436	0.022761	0.038023
H	-0.511877	3.788254	0.017385
H	2.218027	2.390274	0.021119
H	-2.949447	2.821654	0.024114
H	-0.927792	-2.607073	-0.811016
H	1.807622	-1.925628	0.042079
C	3.516733	0.057247	0.031187
N	4.649613	-0.052551	0.030885
H	-0.926144	-2.598244	0.907291

Isomer 10

C	2.018801	-0.379753	-0.040310
C	0.665935	-0.097663	-0.039856
C	0.149636	1.199780	-0.038697
C	-0.075062	-1.301720	-0.040752
C	2.448219	1.968482	-0.038426
C	-3.384881	0.201254	-0.038992
C	2.942830	0.638952	-0.039615
C	1.105519	2.261693	-0.037971
C	-1.238391	1.275139	-0.038405
C	-2.023773	0.079038	-0.039304
C	-4.652321	0.320843	-0.038685
C	-1.440270	-1.238673	-0.040518
H	2.973526	-2.327718	-0.042092
H	3.165689	2.775978	-0.037868
H	4.010458	0.462310	-0.039937
H	0.775329	3.291411	-0.037069
H	-1.752628	2.228082	-0.037503
H	-5.208071	0.373845	-0.974383
H	-5.207761	0.372216	0.897290
H	-2.084679	-2.107495	-0.041175
C	0.919579	-2.351127	-0.041786
N	2.108094	-1.799179	-0.041511
H	0.790424	-3.423717	-0.042662

Isomer 11

C	-1.770356	0.493560	0.039212
C	-0.733776	-0.495508	0.041554
C	0.602648	-0.014794	0.040622
C	-0.681472	-1.873487	0.044608
C	-1.441595	1.829600	0.036136
C	2.363957	1.654923	0.037131
C	-0.080545	2.259522	0.035324
C	0.961611	1.356124	0.037569
C	2.853573	-0.742140	0.042637
C	1.516417	-1.057918	0.043064
C	3.261181	0.629100	0.039625
C	0.728279	-2.336794	0.045835
H	-5.245205	-0.347466	0.040720
H	-2.218102	2.582209	0.034285
H	0.113242	3.324866	0.032884
H	-1.522253	-2.554901	0.046027
H	3.614922	-1.511646	0.044556
H	2.696356	2.683551	0.034872
H	4.319736	0.845804	0.039362
H	0.917200	-2.981721	-0.821991
C	-3.150588	0.141352	0.039860
N	-4.256221	-0.126400	0.040341
H	0.916880	-2.977662	0.916748

Isomer 12

C	-0.569770	1.856021	0.040590
C	-0.594272	0.441543	0.038975
C	0.641671	-0.188221	0.038903
C	-1.694880	-0.463073	0.037356
C	0.625126	2.569845	0.042014
C	2.892728	-0.411703	0.039771
C	1.862343	1.932374	0.041944
C	1.875984	0.524839	0.040351
C	0.850504	-1.573324	0.037402
C	-0.234741	-2.405335	0.035855
C	2.342903	-1.792922	0.037904
C	-1.528544	-1.827358	0.035850
H	-1.494740	2.418960	0.040750
H	0.584432	3.649339	0.043205
H	2.778942	2.507175	0.043077
H	3.953942	-0.195091	0.040537
H	-0.142863	-3.482325	0.034646
H	2.710167	-2.350100	-0.832275
H	2.709287	-2.352097	0.907173
H	-2.396255	-2.472091	0.034648
C	-4.082345	0.394526	0.037123
N	-3.006019	0.031581	0.037250
H	-5.106142	0.737974	0.037003

Isomer 13

C	0.659771	-1.831613	0.029812
C	0.615330	-0.409815	0.028092
C	-0.655734	0.186196	0.028054
C	1.667250	0.528910	0.026352
C	-0.515969	-2.528224	0.031318
C	-2.963400	0.493616	0.028911
C	-1.795372	-1.890919	0.031255
C	-1.853997	-0.524992	0.029597
C	-0.869038	1.587929	0.026410
C	0.216236	2.475158	0.024697
C	-2.256289	1.786141	0.026908
C	1.492073	1.923343	0.024690
H	5.060990	-0.630742	0.026016
H	-0.486425	-3.608502	0.032615
H	-2.686563	-2.503949	0.032504
H	-3.639629	0.440236	0.894618
H	0.085241	3.548394	0.023428
H	-3.640525	0.438062	-0.835960
H	-2.758251	2.746407	0.025962
H	2.357117	2.571152	0.023407
C	3.022016	0.051804	0.026233
N	4.097505	-0.315184	0.026127
H	1.600443	-2.365000	0.029943

Isomer 14

C	0.908744	1.937624	-0.029930
C	0.873379	0.555258	-0.028822
C	-0.362287	-0.075475	-0.029492
C	1.861559	-0.549248	-0.026934
C	-0.301708	2.673559	-0.031751
C	-2.715996	-0.157292	-0.031644
C	-1.523384	2.054231	-0.032430
C	-1.585370	0.623181	-0.031259
C	-1.311168	-2.277859	-0.028525
C	-0.209641	-1.497769	-0.028141
C	-2.644550	-1.628515	-0.030315
C	1.225323	-1.757885	-0.026534
H	5.406251	-0.248879	-0.023856
H	-0.247520	3.752307	-0.032606
H	-2.436006	2.634583	-0.033822
H	-3.242551	-2.006644	-0.877533
H	-1.270779	-3.359839	-0.027560
H	-3.703142	0.292151	-0.032953
H	-3.244087	-2.005170	0.816478
H	1.694223	-2.730139	-0.025256
C	3.262856	-0.426443	-0.025718
N	4.397958	-0.323852	-0.024744
H	1.843537	2.484156	-0.029454

Isomer 15

C	-0.898555	1.414684	0.038194
C	-0.680039	0.027209	0.037415
C	0.554125	-0.572638	0.037029
C	-1.907841	-0.649044	0.037146
C	0.136188	2.314669	0.038620
C	2.963229	-0.065188	0.037322
C	1.422867	1.753300	0.038258
C	1.662564	0.355448	0.037499
C	0.543911	-1.995105	0.036267
C	-0.652039	-2.681931	0.035980
C	4.177682	-0.446493	0.037201
C	-1.909576	-2.031811	0.036417
H	-2.794930	2.454183	0.038926
H	0.001835	3.386836	0.039211
H	2.279910	2.413207	0.038589
H	4.711573	-0.610831	0.972982
H	-0.632609	-3.762257	0.035400
H	4.711700	-0.609913	-0.898667
H	1.472562	-2.549553	0.035906
H	-2.821217	-2.613879	0.036179
C	-2.897056	0.384349	0.037802
N	-2.302158	1.569178	0.038396
H	-3.975842	0.303690	0.037859

7 Fundamental transitions - harmonic vs anharmonic calculations

Table S11: Comparison between Harmonic (ω) and Anharmonic (ν) frequencies for Global Minima candidate of phenanthridine^{2+}.

**Mode**	ω_{harm} (cm^{-1})	ν_{anharm} (cm^{-1})	**Ratio** (ν/ω)
0	85.08	89.79	1.0553
1	130.36	130.04	0.9975
2	179.78	176.83	0.9836
3	192.15	66.26	0.3448
4	227.00	230.00	1.0132
5	294.23	293.61	0.9979
6	384.19	303.79	0.7907
7	409.55	404.23	0.9870
8	420.67	424.18	1.0083
9	448.45	446.88	0.9965
10	466.42	467.19	1.0016
11	514.57	520.68	1.0119
12	524.41	519.57	0.9908
13	532.37	509.12	0.9563
14	582.56	579.82	0.9953
15	597.15	591.87	0.9912
16	613.55	606.88	0.9891
17	641.15	646.36	1.0081
18	677.71	664.70	0.9808
19	703.97	696.54	0.9894

Continued on next page

Table S11 – Continued from previous page

Mode	ω_{harm} (cm^{-1})	ν_{anharm} (cm^{-1})	Ratio (ν/ω)
20	761.04	747.49	0.9822
21	761.36	753.40	0.9896
22	789.67	793.67	1.0051
23	852.63	842.73	0.9884
24	857.56	866.62	1.0106
25	940.48	929.39	0.9882
26	942.60	932.23	0.9890
27	951.67	942.99	0.9909
28	967.37	951.46	0.9836
29	1000.54	984.43	0.9839
30	1036.00	1021.61	0.9861
31	1063.01	1039.49	0.9779
32	1067.92	1050.19	0.9834
33	1097.92	1082.73	0.9862
34	1112.27	1098.50	0.9876
35	1167.53	1090.47	0.9340
36	1170.80	1152.43	0.9843
37	1217.50	1201.43	0.9868
38	1247.72	1228.12	0.9843
39	1253.04	1230.51	0.9820
40	1268.01	1243.15	0.9804
41	1318.16	1285.27	0.9751
42	1359.64	1228.32	0.9034
43	1373.58	1338.15	0.9742

Continued on next page

Table S11 – Continued from previous page

**Mode**	ω_{harm} **(cm^{-1})**	ν_{anharm} **(cm^{-1})**	**Ratio** (ν/ω)
44	1408.25	1382.25	0.9815
45	1431.81	1400.77	0.9783
46	1452.48	1420.12	0.9777
47	1474.03	1444.33	0.9799
48	1533.44	1613.10	1.0519
49	1544.32	1510.22	0.9779
50	1628.20	1595.43	0.9799
51	1666.15	1636.08	0.9819
52	1688.21	1754.77	1.0394
53	2386.74	2345.24	0.9826
54	3046.24	2910.82	0.9555
55	3074.00	2924.03	0.9512
56	3206.76	3083.10	0.9614
57	3219.51	3101.28	0.9633
58	3221.60	3095.30	0.9608
59	3222.68	3307.56	1.0263
60	3226.09	3101.41	0.9614
61	3237.51	3117.78	0.9630
62	3645.65	3483.52	0.9555
**Average Intrinsic Scaling Factors**			
**Low Freq.** ($<$ 2000 cm^{-1})	-	-	**0.9731** ($\pm$0.0939)
**High Freq.** ($>$ 2000 cm^{-1})	-	-	**0.9681** ($\pm$0.0210)

Table S12: Comparison between Harmonic (ω) and Anharmonic (ν) frequencies for second most stable candidate of phenanthridine^{2+}.

**Mode**	ω_{harm} **(cm^{-1})**	ν_{anharm} **(cm^{-1})**	**Ratio (ν/ω)**
0	84.23	77.48	0.9199
1	124.78	123.93	0.9932
2	168.51	167.15	0.9919
3	177.94	178.79	1.0048
4	238.59	239.63	1.0044
5	292.30	292.65	1.0012
6	388.35	384.49	0.9901
7	400.08	400.82	1.0019
8	423.84	417.80	0.9857
9	455.31	457.03	1.0038
10	478.54	467.50	0.9769
11	502.37	486.29	0.9680
12	526.91	519.56	0.9861
13	533.04	529.75	0.9938
14	568.98	570.80	1.0032
15	592.16	590.11	0.9965
16	633.46	629.84	0.9943
17	662.93	672.39	1.0143
18	677.88	666.39	0.9831
19	687.14	674.82	0.9821
20	752.46	751.87	0.9992
21	764.41	757.12	0.9905
22	787.14	784.12	0.9962

Continued on next page

Table S12 – Continued from previous page

**Mode**	ω_{harm} (cm^{-1})	ν_{anharm} (cm^{-1})	**Ratio** (ν/ω)
23	817.28	801.32	0.9805
24	859.90	885.43	1.0297
25	898.96	887.45	0.9872
26	947.79	932.27	0.9836
27	978.69	963.30	0.9843
28	989.57	976.80	0.9871
29	1015.35	1010.39	0.9951
30	1031.16	1027.76	0.9967
31	1050.34	1022.82	0.9738
32	1060.13	1053.39	0.9936
33	1098.01	1087.27	0.9902
34	1141.72	1121.00	0.9819
35	1169.23	1140.92	0.9758
36	1173.79	1157.23	0.9859
37	1226.21	1214.57	0.9905
38	1246.68	1222.52	0.9806
39	1255.52	1235.08	0.9837
40	1265.86	1238.12	0.9781
41	1309.35	1281.24	0.9785
42	1354.68	1313.95	0.9699
43	1379.58	1349.20	0.9780
44	1410.54	1386.43	0.9829
45	1418.78	1392.39	0.9814
46	1466.16	1435.99	0.9794

Continued on next page

Table S12 – Continued from previous page

**Mode**	ω_{harm} **(cm^{-1})**	ν_{anharm} **(cm^{-1})**	**Ratio** (ν/ω)
47	1486.58	1465.63	0.9859
48	1527.58	1483.12	0.9709
49	1547.92	1514.12	0.9782
50	1634.41	1592.88	0.9746
51	1651.61	1623.96	0.9833
52	1684.80	1647.19	0.9777
53	2379.52	2333.51	0.9807
54	3042.38	2909.55	0.9563
55	3068.51	2919.60	0.9515
56	3200.11	3089.11	0.9653
57	3219.75	3114.16	0.9672
58	3222.65	3101.48	0.9624
59	3223.41	3100.30	0.9618
60	3231.89	3096.01	0.9580
61	3236.54	3124.76	0.9655
62	3642.06	3481.15	0.9558
**Average Intrinsic Scaling Factors**			
**Low Freq.** ($<$ 2000 cm^{-1})	-	-	**0.9868** ($\pm$0.0148)
**High Freq.** ($>$ 2000 cm^{-1})	-	-	**0.9624** ($\pm$0.0077)

Table S13: Comparison between Harmonic (ω) and Anharmonic (ν) frequencies for third most stable candidate of phenanthridine^{2+}.

**Mode**	ω_{harm} **(cm^{-1})**	ν_{anharm} **(cm^{-1})**	**Ratio** (ν/ω)
0	79.43	71.31	0.8978
1	121.74	119.39	0.9807
2	174.71	169.01	0.9674
3	193.26	190.85	0.9875
4	220.05	209.34	0.9513
5	296.50	281.54	0.9495
6	375.07	371.82	0.9913
7	421.44	421.51	1.0002
8	421.81	333.32	0.7902
9	454.81	450.04	0.9895
10	468.92	460.79	0.9827
11	524.57	518.15	0.9878
12	527.36	533.08	1.0109
13	542.04	534.31	0.9857
14	580.92	574.47	0.9889
15	611.93	603.12	0.9856
16	631.69	624.23	0.9882
17	664.58	664.45	0.9998
18	674.66	659.01	0.9768
19	680.47	664.07	0.9759
20	709.67	701.26	0.9881
21	763.16	778.21	1.0197
22	783.17	781.74	0.9982

Continued on next page

Table S13 – Continued from previous page

**Mode**	ω_{harm} **(cm^{-1})**	ν_{anharm} **(cm^{-1})**	**Ratio** (ν/ω)
23	838.92	854.00	1.0180
24	850.95	839.00	0.9859
25	878.79	892.36	1.0154
26	955.92	940.90	0.9843
27	963.91	964.59	1.0007
28	992.45	972.61	0.9800
29	1017.72	1015.87	0.9982
30	1035.90	1023.92	0.9884
31	1059.49	1026.63	0.9690
32	1070.53	971.25	0.9073
33	1089.55	1078.69	0.9900
34	1154.12	1136.95	0.9851
35	1175.09	1149.30	0.9781
36	1196.24	1176.99	0.9839
37	1212.25	1182.22	0.9752
38	1219.97	1201.41	0.9848
39	1247.56	1234.25	0.9893
40	1259.38	1236.96	0.9822
41	1307.57	1276.12	0.9759
42	1369.82	1341.33	0.9792
43	1389.98	1346.19	0.9685
44	1395.21	1369.89	0.9819
45	1431.32	1399.70	0.9779
46	1461.55	1431.30	0.9793

Continued on next page

Table S13 – Continued from previous page

**Mode**	ω_{harm} **(cm^{-1})**	ν_{anharm} **(cm^{-1})**	**Ratio** (ν/ω)
47	1492.22	1279.13	0.8572
48	1525.30	1463.63	0.9596
49	1541.53	1497.01	0.9711
50	1630.91	1595.19	0.9781
51	1669.77	1642.46	0.9836
52	1674.37	1641.36	0.9803
53	2386.69	2336.35	0.9789
54	3050.23	2919.44	0.9571
55	3081.67	2934.43	0.9522
56	3206.98	3079.91	0.9604
57	3220.49	3097.47	0.9618
58	3223.54	3120.70	0.9681
59	3228.72	3102.53	0.9609
60	3234.90	3111.44	0.9618
61	3235.74	3115.45	0.9628
62	3636.42	3472.16	0.9548
**Average Intrinsic Scaling Factors**			
**Low Freq.** ($<$ 2000 cm^{-1})	-	-	**0.9755** ($\pm$0.0371)
**High Freq.** ($>$ 2000 cm^{-1})	-	-	**0.9619** ($\pm$0.0071)

Table S14: Comparison between Harmonic (ω) and Anharmonic (ν) frequencies for fourth most stable candidate of phenanthridine^{2+}.

**Mode**	ω_{harm} **(cm^{-1})**	ν_{anharm} **(cm^{-1})**	**Ratio** (ν/ω)
0	83.06	87.36	1.0516
1	130.98	131.54	1.0043
2	176.74	177.57	1.0047
3	209.10	211.14	1.0098
4	213.75	213.56	0.9991
5	288.49	289.32	1.0029
6	383.91	380.48	0.9911
7	407.69	410.10	1.0059
8	411.69	405.95	0.9861
9	457.01	454.41	0.9943
10	461.27	455.87	0.9883
11	516.12	519.07	1.0057
12	523.41	518.83	0.9912
13	528.34	497.59	0.9418
14	590.04	587.58	0.9958
15	593.85	595.90	1.0034
16	617.63	617.09	0.9991
17	632.96	626.68	0.9901
18	686.36	671.70	0.9787
19	707.24	708.85	1.0023
20	732.39	702.23	0.9588
21	757.18	757.28	1.0001
22	792.66	787.68	0.9937

Continued on next page

Table S14 – Continued from previous page

**Mode**	ω_{harm} **(cm^{-1})**	ν_{anharm} **(cm^{-1})**	**Ratio** (ν/ω)
23	835.75	837.07	1.0016
24	845.56	837.24	0.9902
25	944.59	937.83	0.9929
26	948.47	935.37	0.9862
27	962.80	945.46	0.9820
28	974.10	965.20	0.9909
29	998.44	984.21	0.9858
30	1016.39	1005.55	0.9893
31	1056.42	1020.44	0.9659
32	1057.49	1046.14	0.9893
33	1071.85	1053.29	0.9827
34	1149.05	1117.41	0.9725
35	1152.14	1135.34	0.9854
36	1159.46	1140.55	0.9837
37	1202.07	1187.00	0.9875
38	1234.01	1212.92	0.9829
39	1251.37	1227.92	0.9813
40	1299.61	1281.20	0.9858
41	1311.88	1281.34	0.9767
42	1330.88	1299.81	0.9767
43	1366.87	1335.92	0.9774
44	1391.68	1359.67	0.9770
45	1412.86	1386.20	0.9811
46	1464.66	1439.49	0.9828

Continued on next page

Table S14 – Continued from previous page

**Mode**	ω_{harm} **(cm^{-1})**	ν_{anharm} **(cm^{-1})**	**Ratio (ν/ω)**
47	1480.99	1448.50	0.9781
48	1511.52	1473.85	0.9751
49	1549.22	1514.97	0.9779
50	1628.73	1592.13	0.9775
51	1648.68	1612.30	0.9779
52	1685.96	1654.97	0.9816
53	2390.17	2349.33	0.9829
54	3024.97	2888.56	0.9549
55	3046.15	2893.50	0.9499
56	3205.34	3085.75	0.9627
57	3214.93	3101.07	0.9646
58	3222.64	3097.89	0.9613
59	3223.82	3105.67	0.9634
60	3227.30	3103.25	0.9616
61	3237.43	3111.05	0.9610
62	3631.55	3468.87	0.9552
**Average Intrinsic Scaling Factors**			
**Low Freq.** (< 2000 cm^{-1})	-	-	**0.9882** (±0.0152)
**High Freq.** (> 2000 cm^{-1})	-	-	**0.9617** (±0.0083)

Table S15: Comparison between Harmonic (ω) and Anharmonic (ν) frequencies for less stable candidate of phenanthridine^{2+}.

**Mode**	ω_{harm} **(cm^{-1})**	ν_{anharm} **(cm^{-1})**	**Ratio** (ν/ω)
0	80.85	90.39	1.1180
1	164.70	163.24	0.9912
2	214.14	214.12	0.9999
3	284.40	296.25	1.0417
4	309.82	324.11	1.0461
5	399.32	382.31	0.9574
6	408.46	412.96	1.0110
7	433.57	444.39	1.0250
8	487.43	480.18	0.9851
9	504.31	489.81	0.9712
10	537.49	529.05	0.9843
11	562.81	573.39	1.0188
12	563.51	549.46	0.9751
13	661.67	669.81	1.0123
14	665.22	657.50	0.9884
15	684.30	698.36	1.0205
16	688.83	677.85	0.9841
17	763.90	852.84	1.1164
18	783.22	769.09	0.9820
19	794.63	803.66	1.0114
20	835.55	821.98	0.9838
21	842.65	833.41	0.9890
22	862.65	901.97	1.0456

Continued on next page

Table S15 – Continued from previous page

**Mode**	ω_{harm} **(cm^{-1})**	ν_{anharm} **(cm^{-1})**	**Ratio** (ν/ω)
23	914.56	904.36	0.9888
24	916.77	898.63	0.9802
25	957.71	943.08	0.9847
26	972.49	965.90	0.9932
27	1008.96	996.34	0.9875
28	1011.41	1001.94	0.9906
29	1018.17	1003.70	0.9858
30	1053.44	1050.04	0.9968
31	1062.01	1036.10	0.9756
32	1100.18	1072.79	0.9751
33	1131.59	1113.37	0.9839
34	1172.82	1159.96	0.9890
35	1185.16	1157.10	0.9763
36	1208.58	1175.41	0.9726
37	1229.64	1210.19	0.9842
38	1248.58	1226.19	0.9821
39	1296.57	1273.75	0.9824
40	1330.86	1306.58	0.9818
41	1345.84	1301.04	0.9667
42	1386.30	1356.91	0.9788
43	1392.05	1349.81	0.9697
44	1406.67	1373.51	0.9764
45	1452.14	1414.29	0.9739
46	1465.71	1436.91	0.9804

Continued on next page

Table S15 – Continued from previous page

Mode	ω_{harm} (cm^{-1})	ν_{anharm} (cm^{-1})	Ratio (ν/ω)
47	1499.05	1450.53	0.9676
48	1533.91	1587.78	1.0351
49	1550.92	1514.18	0.9763
50	1591.71	1556.62	0.9780
51	1627.91	1594.88	0.9797
52	1712.67	1742.92	1.0177
53	1850.74	1812.54	0.9794
54	3060.13	2932.70	0.9584
55	3095.45	2950.22	0.9531
56	3213.72	3090.78	0.9617
57	3222.72	3104.69	0.9634
58	3227.26	3104.79	0.9620
59	3247.70	3290.95	1.0133
60	3252.22	3126.05	0.9612
61	3268.00	3144.10	0.9621
62	3535.08	3382.38	0.9568
**Average Intrinsic Scaling Factors**			
**Low Freq.** (< 2000 cm^{-1})	-	-	**0.9953** (±0.0313)
**High Freq.** (> 2000 cm^{-1})	-	-	**0.9658** (±0.0171)
